# Comparative RNA-Seq analysis on the regulation of cucumber sex differentiation under different ratios of blue and red light

**DOI:** 10.1186/s40529-018-0237-7

**Published:** 2018-09-10

**Authors:** Jiali Song, Yiting Zhang, Shiwei Song, Wei Su, Riyuan Chen, Guangwen Sun, Yanwei Hao, Houcheng Liu

**Affiliations:** College of Horticulture, South China Agricultural University, Guangzhou, 510642 China

**Keywords:** RNA-seq, Auxin, Cucumber, Flower, Sex differentiation

## Abstract

Cucumber (*Cucumis sativus* L.) is a typical monoecism vegetable with individual male and female flowers, which has been used as a plant model for sex determination. It is well known that light is one of the most important environmental stimuli, which control the timing of the transition from vegetative growth to reproductive development. However, whether light controls sex determination remains elusive. To unravel this problem, we performed high-throughput RNA-Seq analyses, which compared the transcriptomes of shoot apices between R2B1(Red light:Blue light = 2:1)-treated and R4B1(Red light:Blue light = 4:1)-treated cucumber seedlings. Results showed that the higher proportion of blue light in the R2B1 treatment significantly induced the formation of female flowers and accelerated female flowering time in this whole study. The genes related to flowering time, such as *flowering locus T* (*FT*) and *SUPPRESSOR OF OVEREXPRESSION OF CO1* (*SOC1*), were up-regulated after R2B1 treatment. Furthermore, the transcriptome analysis showed that up-regulation and down-regulation of specific DEGs (the differentially expressed genes) were primarily the result of plant hormone signal transduction after treatments. The specific DEGs related with auxin formed the highest percentage of DEGs in the plant hormone signal transduction. In addition, the expression levels of transcription factors also changed after R2B1 treatment. Thus, sex differentiation affected by light quality might be induced by plant hormone signal transduction and transcription factors. These results provide a theoretical basis for further investigation of the regulatory mechanism of female flower formation under different light qualities in cucumber seedlings.

**Electronic supplementary material:**

The online version of this article (10.1186/s40529-018-0237-7) contains supplementary material, which is available to authorized users.

## Background

Sex differentiation of flower buds is an important developmental process, which directly affects the product yield in plants. Cucumber (*Cucumis sativus* L.) is a typical monoecious plant with distinct male and female flowers. It has been served as a model system for studying physiological and molecular aspects of sex determination and differentiation in plants (Bai and Xu 2013). In the young floral buds of cucumber, both the stamen primordia and carpel primordia are initiated, with sex determination occurring just after the bisexual stage. Subsequently, male or female flowers are formed and become enlarged due to the selective arrestment of carpel or stamen development, respectively (Bai et al. 2004).

Sex differentiation of cucumber can be affected by phytohormones, such as ethylene and gibberellin. Exogenous ethylene treatment induced female flower formation in the cucumber. The ethylene content in gynoecious cucumbers is found to be higher than that of monoecious plants (Trebitsh et al. 1987; Rudich et al. 1972). Ethylene synthesis genes play an important role in the sex differentiation of the cucumber flowers, such as 1-aminocyclopropane-1-carboxylic acid oxidases (*CsACO2*). Organ-specific overexpression of *CsACO2* driven by the organ-specific promoter P (AP3) significantly affected stamen but not carpel development, acting similarly to natural floral development of female cucumber flowers (Duan et al. 2008). A conserved-residue conversion in *CsACS2* induced the formation of bisexual flowers in the cucumber (Li et al. 2009). Furthermore, ethylene signaling pathway also affects the sex differentiation. *CsETR1*, an ethylene receptor involved in ethylene signaling transduction, had been demonstrated to play a key role in stamen arrest in female cucumber flowers (Wang et al. 2010). Ethylene-responsive gene associated with the formation of female flowers (*ERAF17*), a MADS-box gene, could be induced by ethylene and might be involved in formation of female flowers in cucumbers (Ando et al. 2001). Exogenous gibberellic acid (GA_3_) application promoted the formation of male flowers in gynoecious plants (Pike and Peterson 1969). GA production in andromonoecious cucumbers was higher than that in gynoecious and monoecious plants (Junior et al. 1972). The GA signaling pathway is involved in stamen development in the cucumber (Zhang et al. 2014b; Fei et al. 2004). Cucumber DELLA Homolog (*CsGAIP*) is predominantly expressed in the male specific organs during cucumber flower development and belongs to the DELLA (the negative regulators of the GA action) family. *CsGAIP* inhibited stamen development through transcriptional repression of B-class floral homeotic genes *APETALA3* (*AP3*) and *PISTILLATA* (*PI*) in Arabidopsis (Zhang et al. 2014a). *CsGAMYB1*, a positive regulator involved in the GA signaling pathway, also mediates the sex expression of cucumbers. Knocking out the *CsGAMYB1* gene in cucumbers resulted in decreased ratios of nodes with male to female flowers (Zhang et al. 2014b). The relationship between ethylene and gibberellin in mediating sex differentiation was interpreted recently, with this study finding that gibberellin mediates sex differentiation via ethylene-dependent and ethylene-independent pathways in cucumbers (Zhang et al. 2017). Apart from ethylene and gibberellin, other hormones are also involved in the flower sex differentiation of plant (Song et al. 2013). This includes the abscisic acid (ABA) induction of male flower formation (Zhu et al. 2010); Jasmonic acid (JA) signaling pathway, which might participate in the abortion of male flowers (Acosta et al. 2009); and auxin (IAA) content, which was found to increase during female flower development and decrease during male flower development (Sakata et al. 2010). Ethylene biosynthesis has probably been induced by IAA to promote female flower formation as well (Trebitsh et al. 1987). The IAA induced *CsACS1* gene expression in cucumbers, which suggested that IAA promoted female flower formation through inducing ethylene synthesis (Trebitsh et al. 1997; Mibus and Tatlioglu 2004).

Environmental cues, such as temperature, photoperiod, nutrition, also affected sex determination in many species (Golenberg and West 2013; Korpelainen 1998). Light is one of the major external factors that influence plant growth and development (Chen et al. 2004). Plants respond to light through mediating light receptors. Until now, known photoreceptors were divided into three classes: the UV-B (280–320 nm light) photoreceptors; the red/far-red reversible photoreceptors, known as the phytochromes PhyA–PhyE (Mathews and Sharrock 1997) and blue UVA photoreceptors. These blue UVA photoreceptors have three classes, which are the cryptochromes (CRY1, CRY2 and CRY3) (Mathews and Sharrock 1997; Brudler et al. 2003), phototropins (PHOT1 and PHOT2) (Briggs et al. 2001) and aureochromes (AUREO1 and AUREO2) (Ishikawa et al. 2009; Takahashi et al. 2007). There is less evidence examining the effects of light on plant sex differentiation. In the gametophytes of the *Her1* mutant, blue light induced male development and red light suppressed male development (Kamachi et al. 2007). Our previous studies showed that light quality affected cucumber flower formation and sex differentiation. In our previous study, we used 4R1B (Red:Blue = 4:1), R2B1 (Red:Blue = 2:1), R6G2B1 (Red:Green:Blue = 6:2:1) ratios supplemental light to treat the cucumber seedlings. Comparing to natural light, R2B1 treatment was the best light quality ratio for improving the female flower formation, location and flowering time, but none of them has effects on male flower formation (our unpublished data). However, the mechanism of how light quality regulates flower sex differentiation still remains unclear. In our study, we aimed to understand the mechanism of light regulating cucumber sexual development. We performed RNA-Seq analyses to compare the transcriptomes of shoot apices between cucumber seedlings after R2B1 treatment and R4B1 treatment. Our results built a foundation for dissecting the molecular mechanism of flower sex differentiation in cucumbers.

## Results

### Effects of different LED light quality treatments on flower formation and flowering time

Comparing to natural light, R2B1 treatment was the best light quality ratio for improving the female flower formation, location and flowering time (Additional file 1: Table S1). After 20 days lighting under R2B1 and R4B1 light treatment, the formation of female flowers occurred mainly at the lower node positions under the R2B1 irradiation, as compared with R4B1 irradiation (Table 1). The number of female flowers in the 15 nodes of the cucumber plant increased markedly and flowered earlier (Table 1). All of these results implied that blue light is responsible for the high number of female flowers, early flowering time and reduction in the first female flower node.

**Table 1:** Effects of different light quality on the formation of female flowers and flowering time

Treatment	First female flower located node	Total female flower numbers in 15 nodes	Days from transplanting to first female flowering
R2B1 (Red light:blue light = 2:1)	7.20 ± 0.23b	2.70 ± 0.13a	21.85 ± 0.17b
R4B1 (Red light:blue light = 4:1)	8.03 ± 0.19a	2.32 ± 0.10b	22.53 ± 0.12a

a,b: Statistically significant variations of mean values at different sampling points (ANOVA, p < 0.05) were indicated with different letters. Means denoted by the same letter did not differ significantly at p < 0.05

### Analysis of differentially expressed genes (DEGs) under treatments with different ratios of blue and red light

**Fig. 1:**
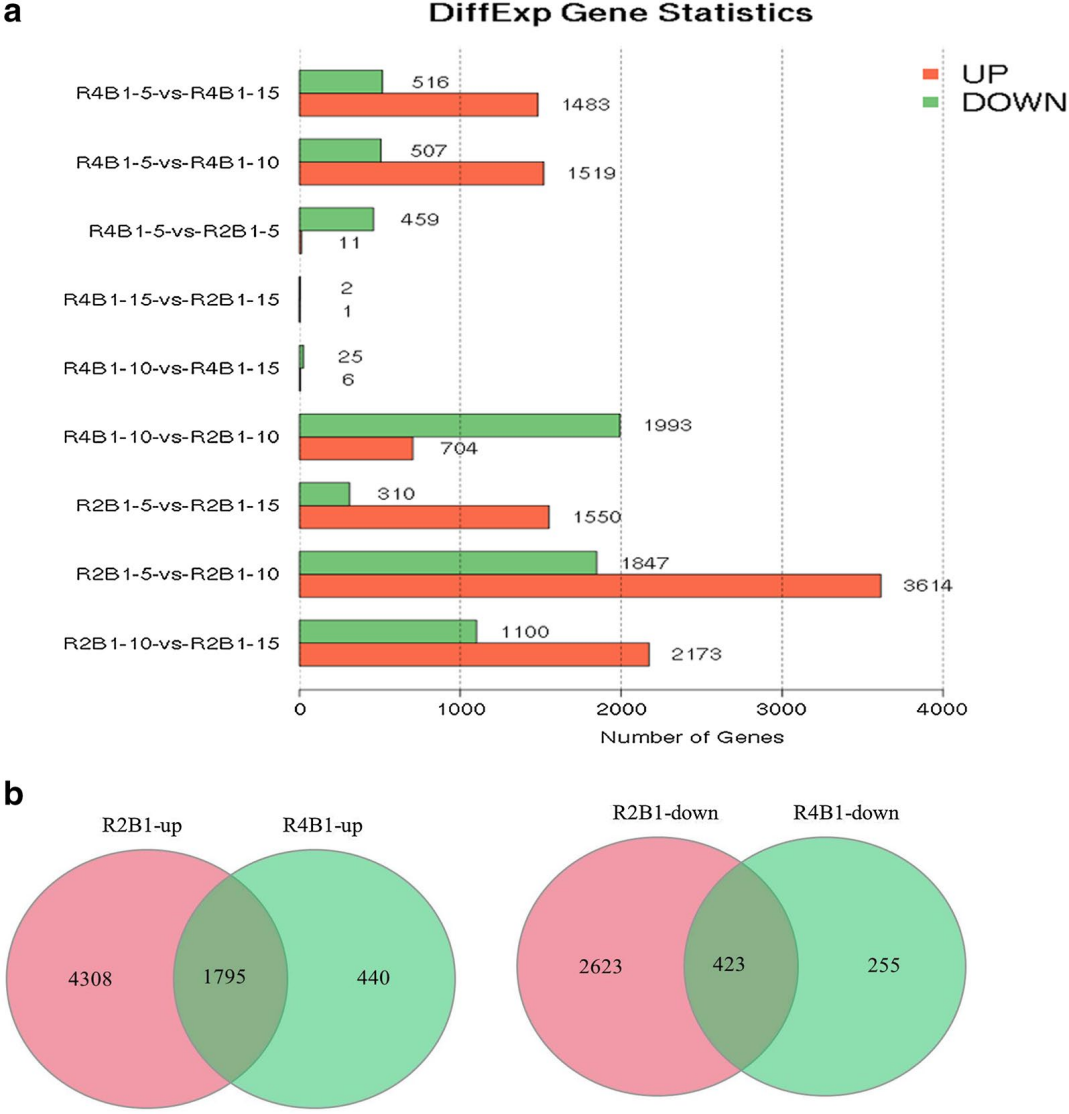
**a** Statistical results of differentially expressed genes (DEGs) among different stages and treatments and **b** Venn diagrams of DEGs that were significantly (left) up-regulated and (right) down-regulated after R2B1 and R4B1 treatment

Based on deep sequencing, 23,248 unigenes were collected in 12 libraries. Genes expressed at differential stages under two treatments were listed in Table 2. After R2B1 treatment, the detected number of genes in the 5 days, 10 days and 15 days library were 18,619, 18,943 and 19,075. The mapping ratios of these libraries according to cucumber genome were 80.09, 81.48 and 82.5%. The detected new genes in these libraries were 603, 609 and 611. The relevant data for R4B1 treatment were shown in Table 2. Using |log2 (fold change)| > 1 and false discovery rate (FDR) < 0.05 as the cut-offs for significance, we identified DEGs at differential stages under two treatments (Fig. 1a). The results showed that there were 470 DEGs including 11 up-regulated genes and 459 down-regulated genes in pairwise R4B1-5-vs-R2B1-5 (5 days library comparison after R4B1 treatment and R2B1 treatment). Furthermore, we also found that 2697 DEGs were differentially expressed, with 704 genes being up-regulated and 1993 genes down-regulated in pairwise R4B1-10-vs-R2B1-10 (10 days library comparison after R4B1 treatment and R2B1 treatment). Moreover, 3 DEGs containing 1 up-regulated gene and 2 down-regulated genes were found in pairwise R4B1-15-vs-R2B1-15 (15 days library comparison after R4B1 treatment and R2B1 treatment). Most DEGs were found in the 10 days samples, which might implicate that the light quality affected flower sex differentiation mostly on the 10th day.

**Table 2:** Statistical results of expressed genes in different libraries

library	Numbers of known genes detected	Numbers of detected new genes
R2B1-5	18,619 (80.09%)	603
R4B1-5	18,831 (81.00%)	614
R2B1-10	18,943 (81.48%)	609
R4B1-10	19,099 (82.15%)	618
R2B1-15	19,075 (82.05%)	611
R4B1-15	19,306 (83.04%)	618

In addition, there were more DEGs in the 5 days, 10 days and 15 days library after R2B1 irradiation compared to R4B1 irradiation. We used a Venn diagram to analyze all these up-regulated or down-regulated DEGs collected from all these treatments at different time points. The results showed that 423 DEGs were found in all three stages after both R4B1 and R2B1 treatment as well as more up-regulated DEGs (1795 DEGs) at all three stages under both treatments (Fig. 1b).

### Gene ontology (GO) analysis of DEGs after different ratios of blue and red light treatments

**Fig. 2:**
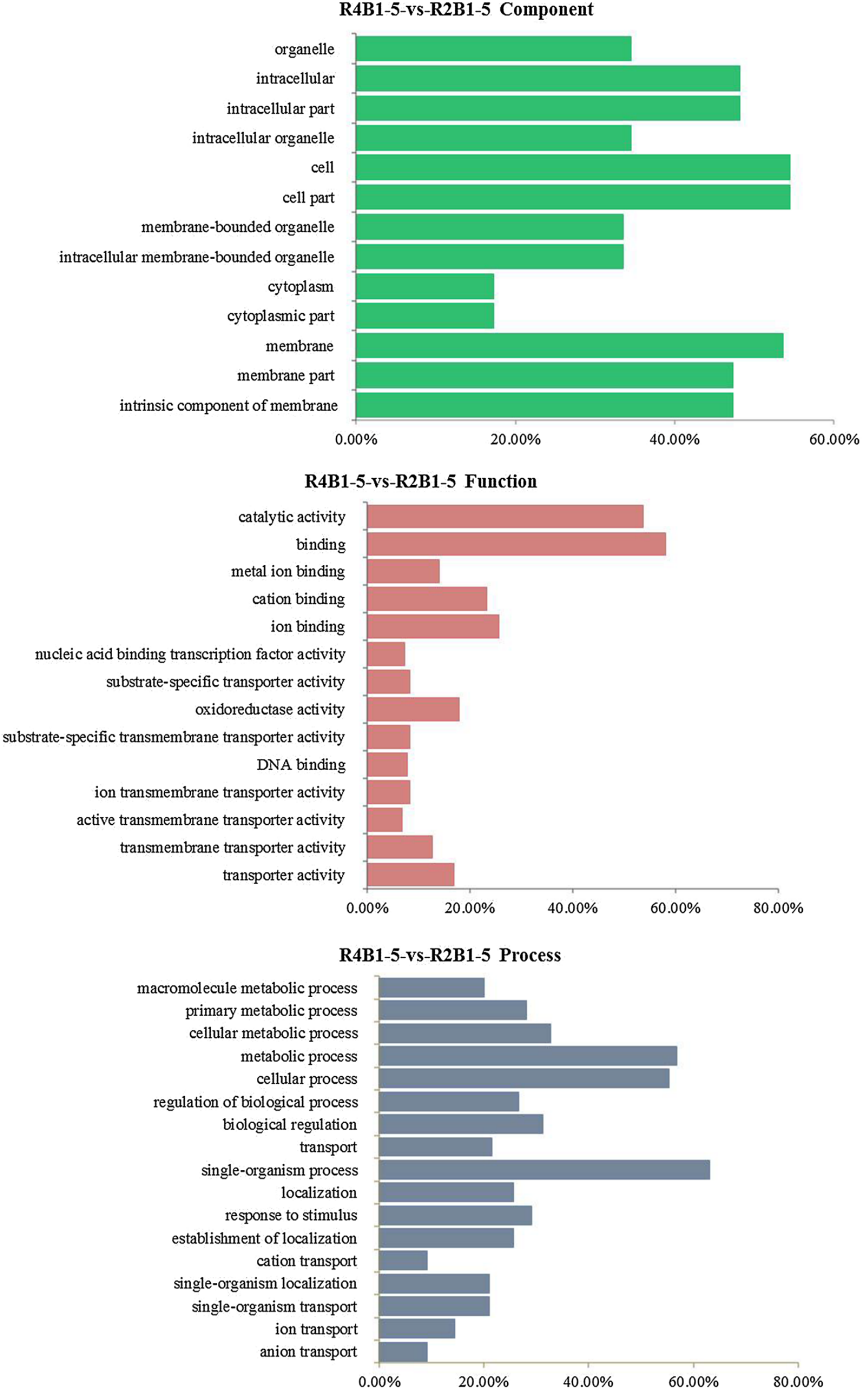
Gene ontology (GO) categories of differently expressed genes (DEGs) in the pairwise R4B1-5-vs-R2B1-5

**Fig. 3:**
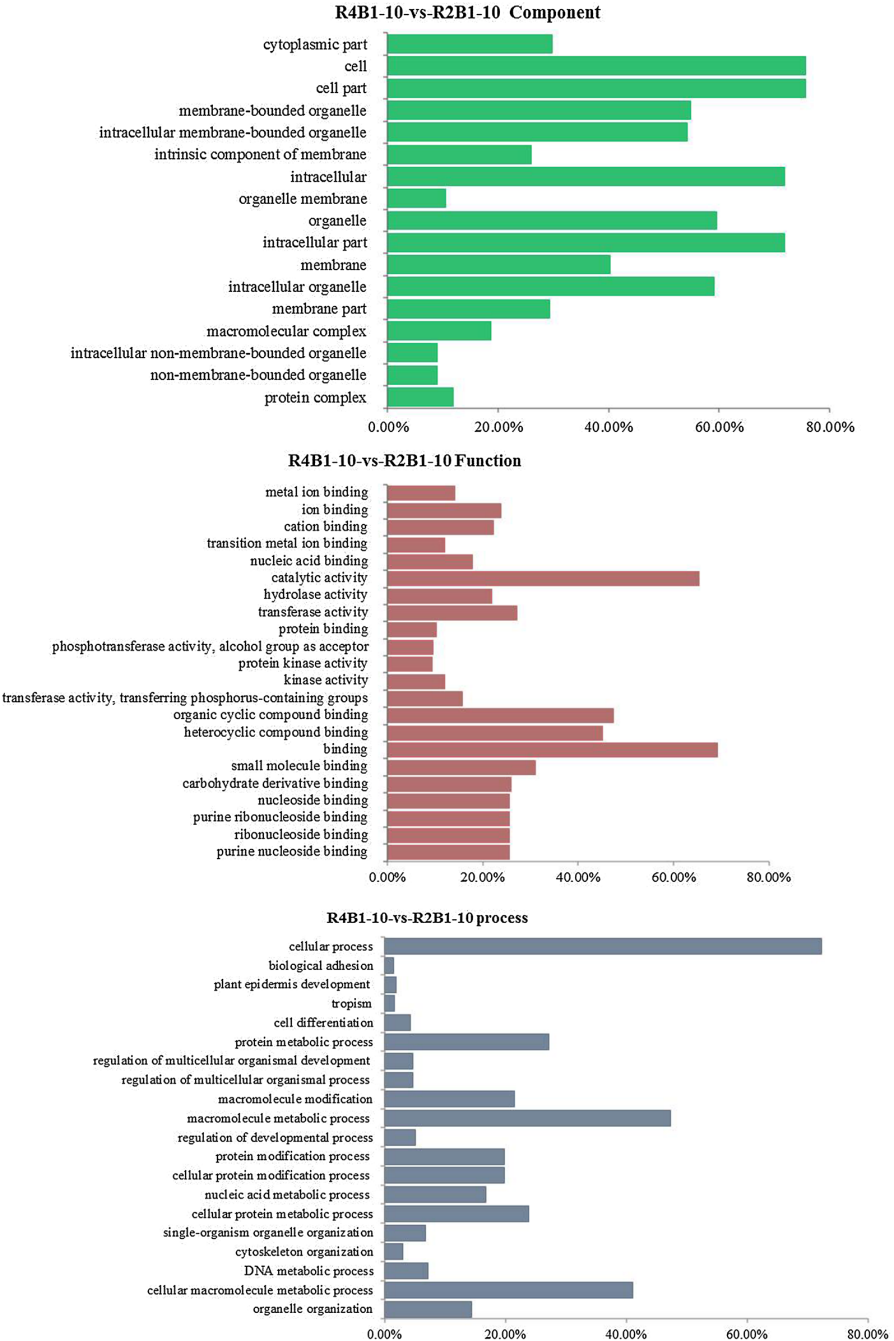
Gene ontology (GO) categories of differently expressed genes (DEGs) in the pairwise R4B1-10-vs-R2B1-10

Gene ontology (GO) mainly includes three categories: molecular function, cellular component and biological process. A total of 110 DEGs in pairwise R4B1-5-vs-R2B1-5 and 745 DEGs in pairwise R4B1-10-vs-R2B1-10 were divided into cell components, which were related to the cell membrane, cytoplasm and organelle (Figs. 2 and 3). In pairwise R4B1-5-vs-R2B1-5, a total of 207 DEGs were classified into molecular functions: catalytic activity, binding, transporter activity and oxidoreductase activity (Fig. 2). A total of 195 DEGs were termed into the biology pathway, which mainly included ion transport, primary metabolic process, localization establishment of localization, biological regulation and response to stimulus (Fig. 2). In pairwise R4B1-10-vs-R2B1-10, 1060 DEGs were termed into molecular functions, which were mainly related to purine nucleoside binding, carbohydrate derivative binding, heterocyclic compound binding, organic cyclic compound binding, catalytic activity, hydrolase activity and transferase activity (Fig. 3). In pairwise R4B1-10-vs-R2B1-10, a total of 1060 genes were classified into the biology pathway. These genes mainly participated in a cellular process, cellular metabolic process, single-organism process, organic substance metabolic process, metabolic process and protein metabolic process (Fig. 3). In pairwise R4B1-15-vs-R2B1-15, only 3 DEGs were found and cannot be classified into any category.

### Kyoto encyclopedia of genes and genomes (KEGG) pathway analysis of DEGs after different ratios of blue and red light treatments

**Fig. 4:**
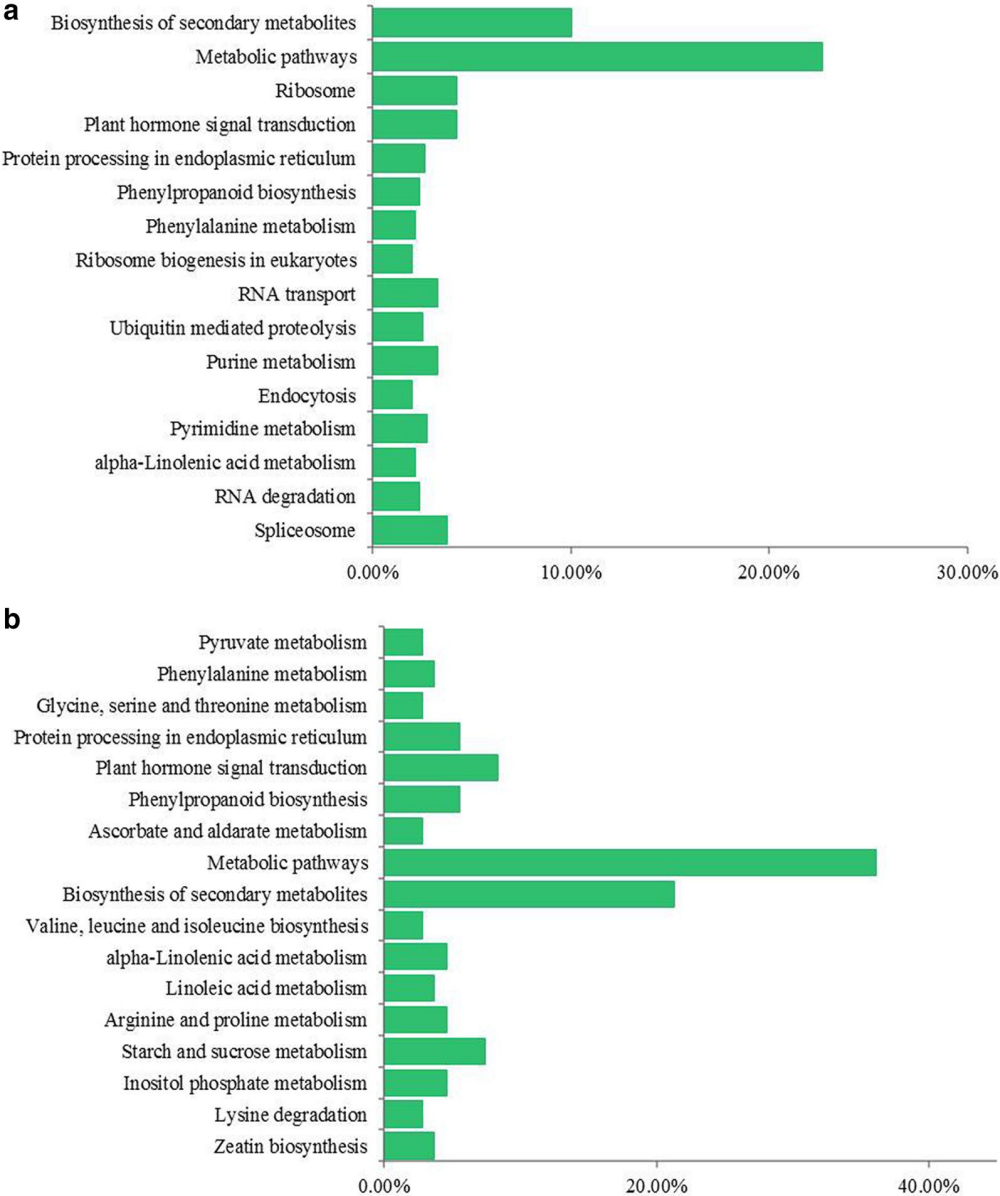
Kyoto encyclopedia of genes and genomes (KEGG) pathway enrichment of differently expressed genes (DEGs) in **a** pairwise R4B1-5-vs-R2B1-5 and **b** pairwise R4B1-10-vs-R2B1-10

To further explore the biological pathways associated with sex differentiation development, DEGs in each comparison were mapped to the reference canonical pathways in the KEGG database. In pairwise R4B1-5-vs-R2B1-5, 108 DEGs were assigned into 52 KEGG pathways. They were significantly enriched in the following pathways: metabolic pathways, biosynthesis of secondary metabolites, starch and sucrose metabolism and plant hormone signal transduction (Fig. 4a). In pairwise R4B1-10-vs-R2B1-10, a total of 798 DEGs were assigned into 106 KEGG pathways. They were significantly enriched in the following pathways: metabolic pathways, biosynthesis of secondary metabolites and plant hormone signal transduction (Fig. 4b).

All specifically up-regulated or down-regulated DEGs after R2B1 and R4B1 treatment were also mapped to the reference canonical pathway in the KEGG database. The results showed that 543 specifically up-regulated DEGs from R2B1 were mostly enriched in the following KEGG pathways: plant hormone signal transduction, secondary protein processing in endoplasmic reticulum, RNA transport and spliceosome pathway (Table 3). A total of 382 specifically down-regulated DEGs from R2B1 were grouped into the following KEGG pathways: biosynthesis of amino acids, protein processing in endoplasmic reticulum and RNA transport pathway (Table 3). The relevant data for R4B1 treatment were listed in Table 3.

**Table 3:** Kyoto encyclopedia of genes and genomes (KEGG) pathway enrichment of significantly up/down-regulated DEGs in R2B1/R4B1

Pathway	DEGs with pathway annotation (543)	P-value	
Plant hormone signal transduction	49	0.000917	Up-regulated DEGs in R2B1
Protein processing in endoplasmic reticulum	40	0.000665	
RNA transport	33	0.012593	
Spliceosome	31	0.078185	
Endocytosis	25	0.010854	
Pyrimidine metabolism	24	0.029783	
RNA degradation	22	0.001054	
Ubiquitin mediated proteolysis	21	0.0133	
Starch and sucrose metabolism	6	0.024124	
Alanine, aspartate and glutamate metabolism	4	0.002149	Up-regulated DEGs in R4B1
Diterpenoid biosynthesis	3	0.005284	
Insulin resistance	3	0.010482	
Biosynthesis of amino acids	34	0.000934	Down-regulated DEGs in R2B1
Protein processing in endoplasmic reticulum	29	0.002805	
RNA transport	29	0.000742	
Spliceosome	28	0.004517	
Purine metabolism	25	0.00642	
Aminoacyl-tRNA biosynthesis	23	0	
Pyrimidine metabolism	22	0.001743	
RNA polymerase	10	0.034698	
Plant hormone signal transduction	16	0.955624	
Ribosome	16	0	Down-regulated DEGs in R4B1
Porphyrin and chlorophyll metabolism	5	0.000222	
Photosynthesis	4	0.002138	

### Auxin plays an important role in the regulation of cucumber sex differentiation under different ratios of blue and red light treatments

**Fig. 5:**
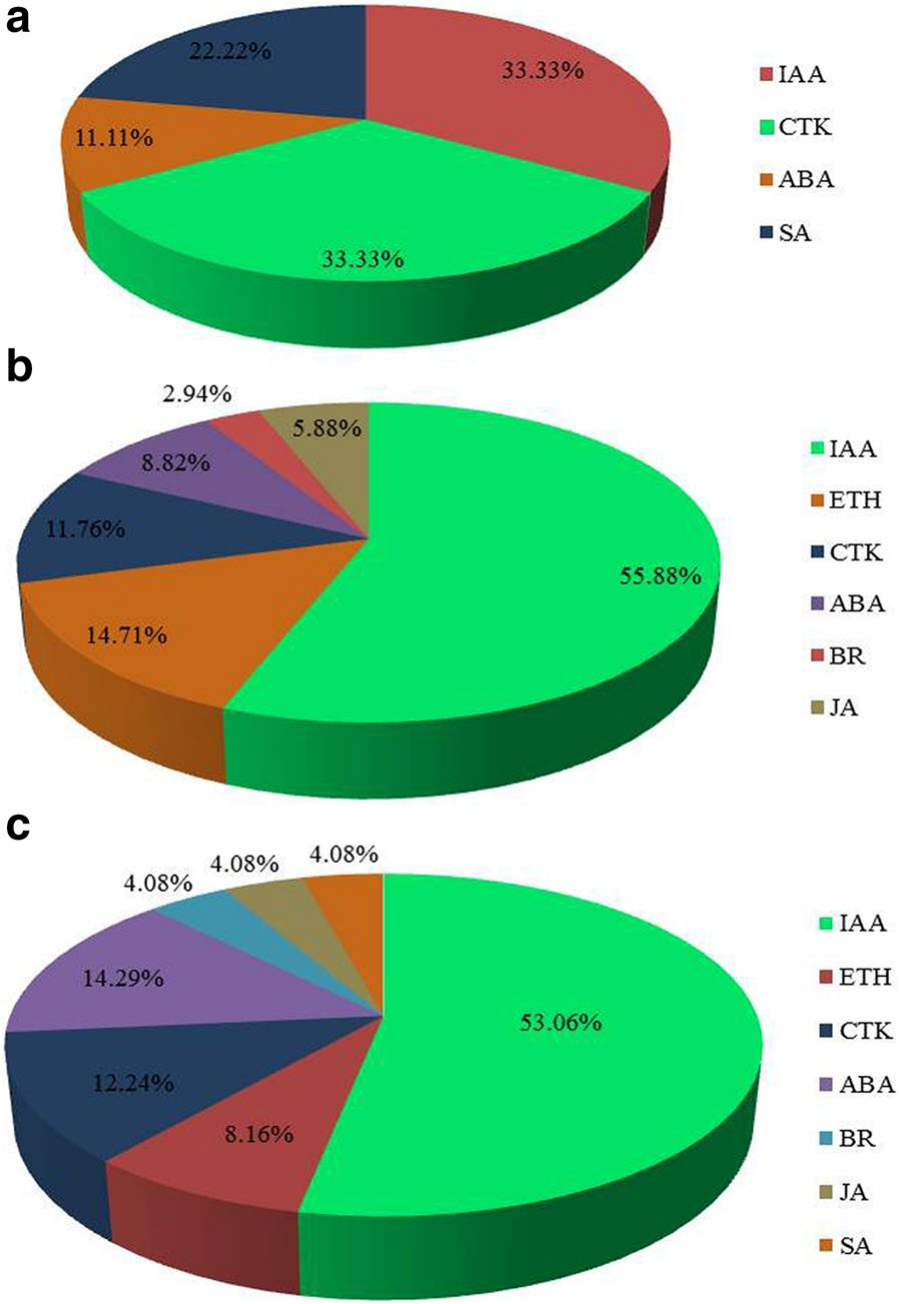
The proportion of differently expressed genes (DEGs) related to plant hormone signaling pathways in **a** pairwise R4B1-5-vs-R2B1-5, **b** pairwise R4B1-10-vs-R2B1-10 and **c** R2B1 library. *IAA* auxin, *CTK* cytokine, *ABA* abscisic acid, *SA* salicylic acid, *ETH* ethylene, *BR* brassinolide, *JA* jasmonic acid

**Fig. 6:**
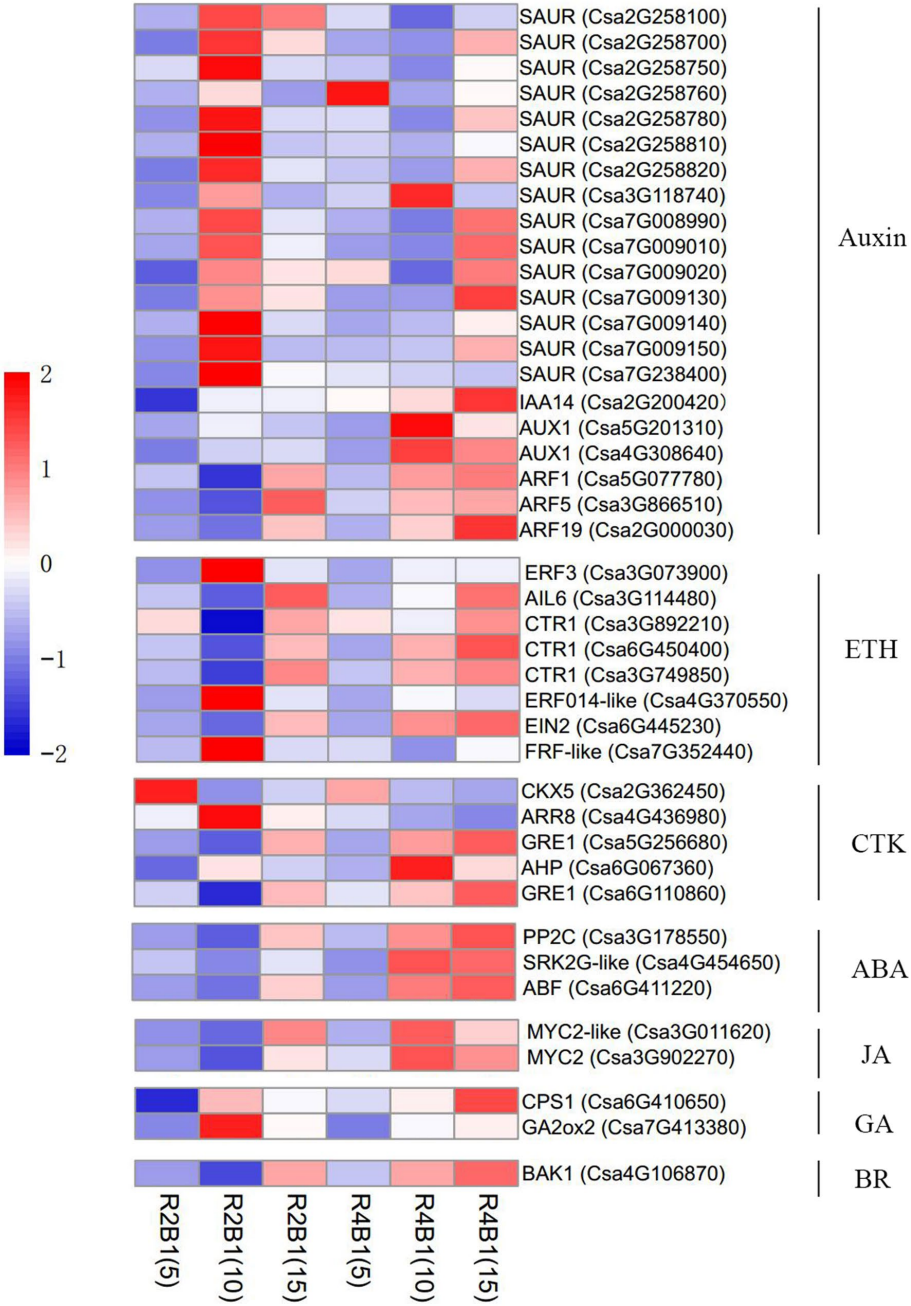
The expression of a number of genes altered in cucumber shoot apices under different light quality treatments: genes involved in plant hormone signaling pathway

Since cucumber sex differentiation is closely related to all types of endogenous hormones, we further explored the role of endogenous hormones regulating the sex differentiation process under different light treatments. Thus, DEGs enriched in plant hormone signal transduction were further analyzed. In pairwise R4B1-5-vs-R2B1-5, 33.33% of the DEGs were related to auxin, 33.33% to cytokines, 22.22% to SA and 11.11% to ABA. In pairwise R4B1-10-vs-R2B1-10, 55.88% of the DEGs were related to auxin, 14.71% to ethylene, 11.76% to CTK, 8.82% to ABA, 5.88% to JA and 2.94% to SA (Fig. 5a, b). In addition, there were 49 up-regulated DEGs related to the plant hormone signaling pathway in R2B1, including 53.06% related to IAA, 8.16% to ETH, 12.24% to CTK and 14.29% to ABA (Fig. 5c). IAA plays an important role during the flower sex differentiation of cucumber seedlings under light irradiation. As most of the DEGs were related to plant hormone, 16 DEGs were found to be involved in auxin biosynthesis and signaling pathway, with most of them showing a higher expression level at R2B1-10 stage (Fig. 6). Five DEGs involved in CTK signaling pathway, eight DEGs in ETH signal transduction pathway and three DEGs in ABA signaling pathway were also identified from the transcriptomes of cucumber shoot apices (Fig. 6). There was no DEGs involved in the GA signal transduction, while only two genes, *CsCPS1* (*Csa6G410650*) and *CsGA2ox2* (*Csa7G413380*), involved in GA biosynthesis were identified. *CsGA2ox2* (*Csa7G413380*) accumulated more transcripts at the R2B1-10 stage, while *CsCPS1* (*Csa6G410650*) expressed more at the R4B1-15 stage (Fig. 6). In conclusion, light regulated cucumber flower sex differentiation through meditating multiple hormone signals: auxin plays an important role; CTK, ABA and ETH function as supplementary regulators; and gibberellin is not involved.

### Validation of RNA-Seq results by real-time quantitative PCR (qRT-PCR)

Twenty-six genes with different expression profiles were randomly selected to verify the RNA-Seq results by qRT-PCR. *α*-*Tubulin* (*TUA*) was used as the internal reference control to standardize the results (Wan et al. 2010). As shown in Additional file 2: Figure S1, twenty-three genes had the same expression pattern. The Pearson correlation coefficient between the RNA-Seq and qRT-PCR data were high, confirming the accuracy of the RNA-Seq data (Additional file 3: Figure S2 and Additional file 4: Figure S3).

### Effect of different ratios of blue and red light treatments on light signal transduction of cucumber seedling leaves

**Fig. 7:**
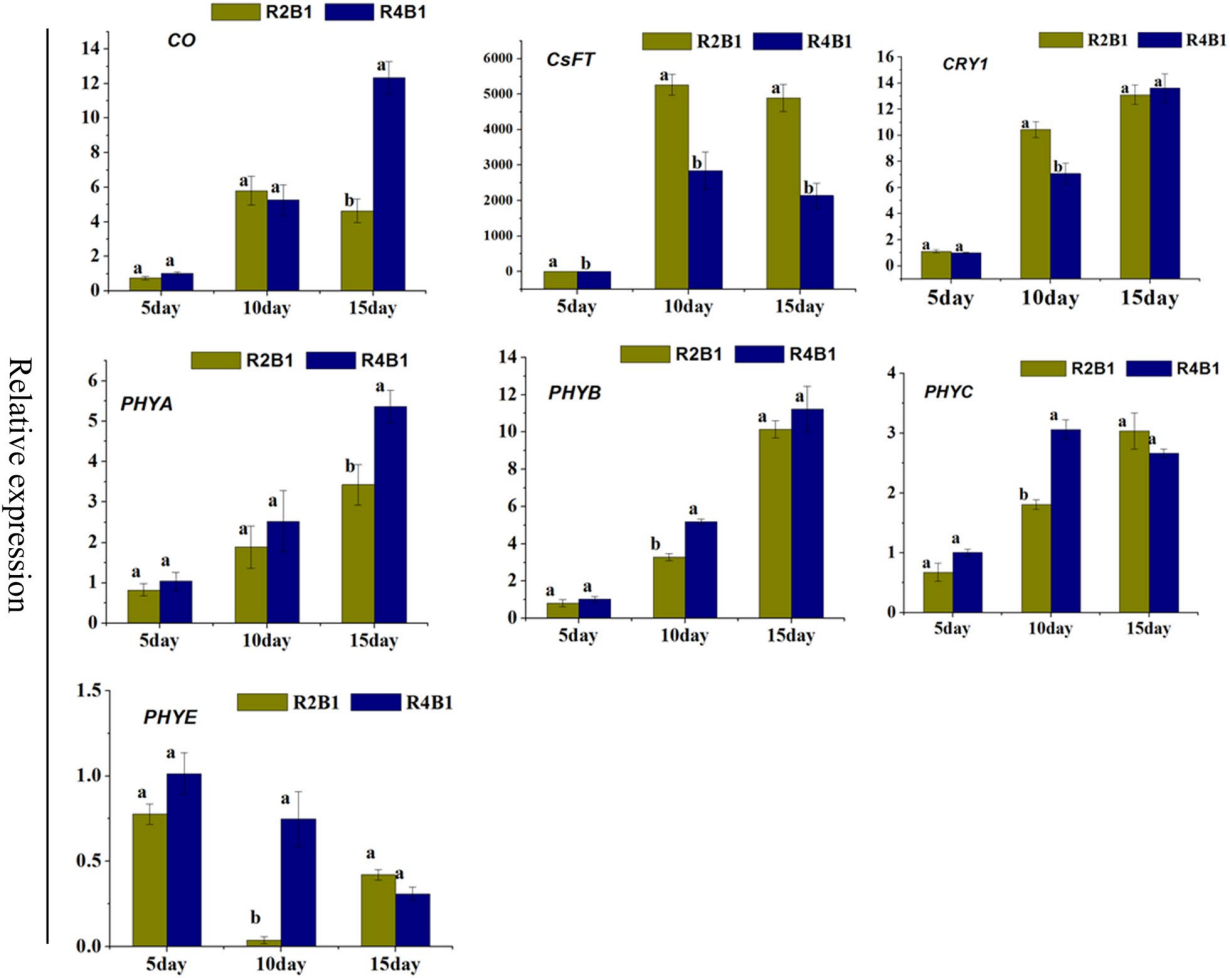
The expression of a number of flowering and light signal transduction related genes were altered in cucumber seedlings leaves under different light quality treatments. Error bars represented standard deviations of the means of three independent replicates. Statistically significant variations of expression and mean values at different sampling points (ANOVA, p < 0.05) were indicated with different letters

Photoperiodic induction affects flower meristem differentiation and number of flowers. As leaves are the site for photoperiodic induction, genes related to light signal transduction and flowering were checked by qRT-PCR on 5-day, 10-day and 15-day cucumber seedling leaves after R2B1 and R4B1 light irradiation. There were two blue light receptors, named CRY1 (Csa3M889670) and CRY2 (Csa3M88160). Only CRY1(Csa3M889670) was found in cucumber leaves. In the 10 days treatment samples, the expression of *CRY1* was higher in R2B1 comparing to R4B1 (Fig. 7). Four red light receptors (PHYA, PHYB, PHYC and PHYE) were found in cucumber leaves. Compared to R2B1 treatment, there was a greater expression of *PHYA* in 10 days samples under R4B1 treatment, while the others showed a higher expression in the 15 days samples after R4B1 treatment (Fig. 7). *CONSTANS* (*CO*) is responsible for turning the light transduction into a flowering signal. It showed a higher expression in 15 days samples after R4B1 treatment than after R2B1 treatment (Fig. 7). *FLOWERING LOCUST* (*FT*) is a key gene in regulating flowering time. *FT* was expressed more in 10 and 15 days samples after R2B1 treatment than after R4B1 treatment (Fig. 7). All of these results suggested that the high proportion of blue light promoted the early flowering of cucumbers by regulating these flowering genes.

### Analysis of differentially expressed genes related to flower sex differentiation of cucumber seedlings under different ratios of blue and red light treatments

**Fig. 8:**
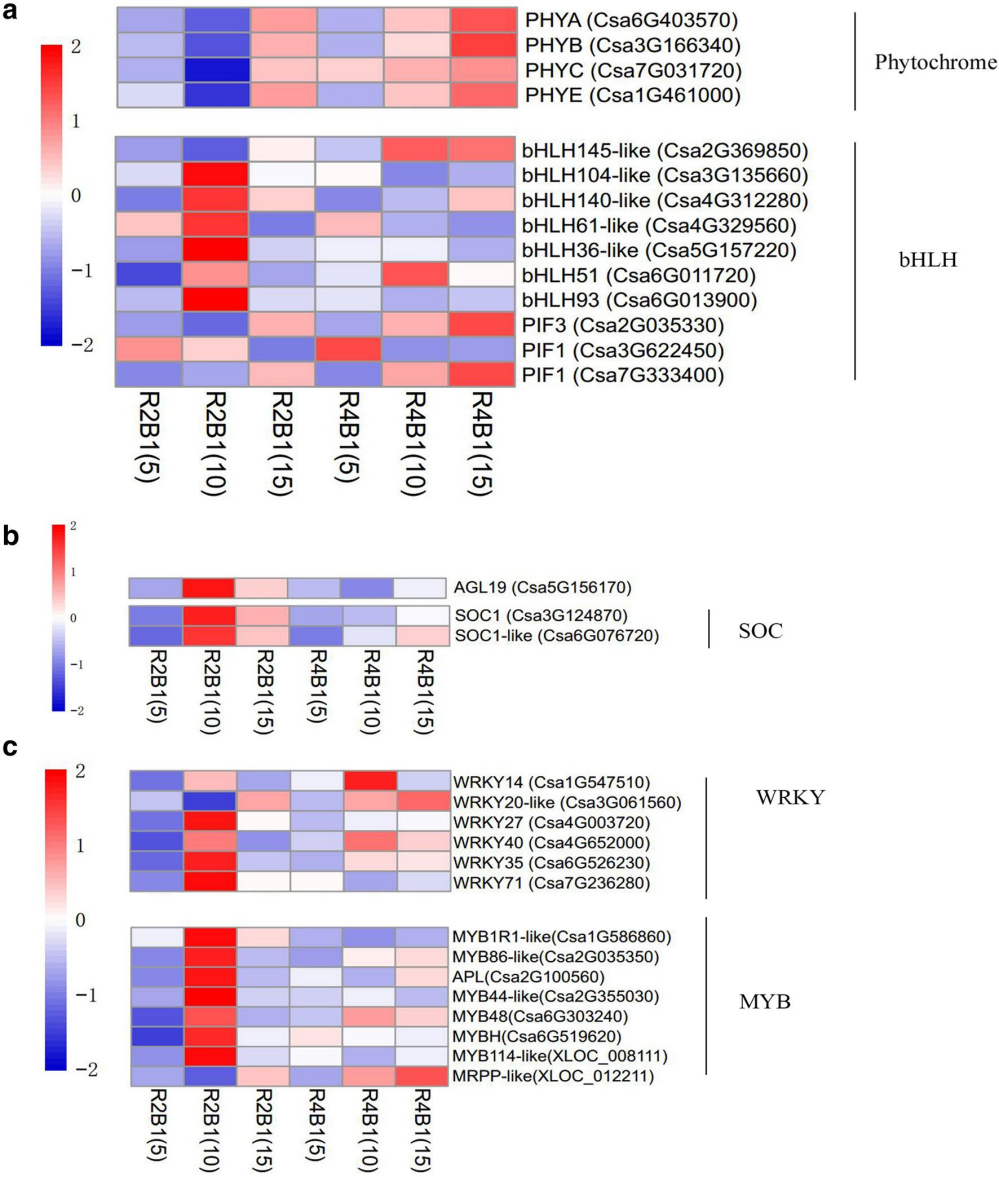
The expression of a number of genes altered in cucumber shoot apices under different light quality treatments: **a** genes involved in light signaling transduction; **b** genes related to flowering time regulation and **c** transcription factors involved in plant hormone and flower organ development

Plant sex differentiation is closely related to endogenous hormone regulation, while flowering time is related to the photoperiod pathway, vernalization and gibberellin pathway. Aiming to identify genes related to flower sex differentiation and promoting flowering under light irradiation, the expression pattern analysis of DEGs was performed in pairwise R4B1-5-vs-R2B1-5, R4B1-10-vs-R2B1-10 and R4B1-15-vs-R2B1-15. Four light receptors (*Csa6G403570* (*PHYA*), *Csa3G166340* (*PHYB*), *Csa7G031720* (*PHYC*) and *Csa1G461000* (*PHYE*)) responding to red light were identified from the shoot apices of cucumber seedlings. These genes showed a higher expression level after R4B1 treatment compared to R2B1 treatment (Fig. 8a). The (basic helix-loop-helix) *bHLH* transcription factor is involved in light signal transduction, hormone signal transduction and flowering pathway. Seven *bHLH* DEGs were identified in the RNA-seq transcriptome, including 6 up-regulated and 1 down-regulated genes in R2B1-10 samples. *PIF*, phytochrome interaction factor, initiates light signal transduction through interacting with the activated phytochrome. *PIF* belongs to *bHLH* family. Its expression levels increased at the R4B1-10 stage, while these levels decreased at the R2B1-10 stage (Fig. 8a). The *WKRY* (transcription factors with WRKYGQK peptide) family transcription factors participate in multiple metabolic pathways. Six differently expressed *WKRY* transcription factors were identified in cucumber shoot apices, including 3 (*Csa4G003720*, *Csa6G526230* and *Csa7G236280*) up-regulated genes and 2 (*Csa1G547510* and *Csa3G061560*) down-regulated genes at R2B1-10 stage (Fig. 8c). The *MYB* transcription factor is related to plant hormone and flower organ development. Nine *MYB* DEGs were identified in shoot apices transcriptome of cucumber seedlings, including 2 new genes (*XLOC_008111* and *XLOC_012211*), 7 (*Csa1G586860*, *Csa2G035350*, *Csa2G100560*, *Csa2G355030*, *Csa6G303240*, *Csa6G519620* and *XLOC_012211*) up-regulated genes and 2 (*Csa4G338950* and *XLOC_012211*) down-regulated genes at the R2B1-10 stage (Fig. 8c) (AGAMOUS [AG]-like MADS box protein) *AGL* and (*SUPPRESSOR OF OVEREXPRESSION OF CO1*) *SOC1* belong to MADS-box gene, which both could regulate flowering time. Seven *AGLs* and two *SOC1* *s* were identified from the shoot apices of cucumber seedlings. *AGL* (*Csa5G156170*) and *SOC1* (*Csa3G124870, Csa6G076720*) accumulated more transcripts at the R2B1-10 stage (Fig. 8b, c).

## Discussion

### Light quality affects cucumber flowering time

Blue light is a strong signal in floral bud formation. In Chrysanthemum, flower budding was formed even after a longer photoperiod than a critical day length under blue light illumination (Jerzy et al. 2011). In petunia plants, floral bud formation and flowering occurred earlier under blue light treatment, while no floral buds were observed under low red irradiance. High red irradiance and temporal switching to blue light during long-term low red irradiance induced floral development (Fukuda et al. 2016). In our experiment, we found that the first female flower opened earlier and occurred mainly at the lower node positions under a higher proportion of blue light irradiance (Table 1). This suggested that blue light promoted flower bud formation and accelerated cucumber flowering.

Cryptochromes (CRY) are flavo-proteins that direct a diverse array of developmental processes in response to blue light in plants (Liu et al. 2016). *CRY1* and *CRY2* function as major blue light receptors regulating blue light-induced de-etiolation and photo-periodic flowering (Guo and Li 1998). In Arabidopsis, *CRY1* and *CRY2* serve both distinct and partially overlapping functions in regulating photomorphogenic responses and photoperiodic flowering. The gain-of-function mutant alleles of *CRY1* exhibited an early flowering phenotype after several days (Exner et al. 2010). In our study, we detected a higher expression of *CRY1* in cucumber seedling leaves after R2B1 treatment than R4B1 treatment (Fig. 7), which was consistent with the earlier flowering phenotype after R2B1 irradiation. The phytochrome (phy) family of sensory photoreceptors (phyA to phyE in *Arabidopsis thaliana*) also responds to inducing floral budding. Low red/far-red ratio promotes flowering in Arabidopsis through *PHYA* inhibiting *PHYB* (Chory 2003; Halliday et al. 2003). In our study, we detected 4 phytochrome orthologous genes in cucumber seedling leaves and shoot apices. Expression analysis showed that *PHYA* accumulated more in leaves at the R4B1-15 stage than R2B1-15 stage (Fig. 7). In comparison, the other genes displayed a higher expression in leaves at R4B1-10 stage than R2B1-10 stage (Fig. 7). Similar results were found in shoot apices, with *PHYA*, *PHYB*, *PHYC*, *PHYE* expressed more in the shoot apices of R4B1-10 sample than R2B1-10 sample (Fig. 7). The expression of *CRY2* and *PHYD* were not detected in both cucumber leaves and shoot apices.

CONSTANS (*CO*) is a key transcription factor regulating flowering time. *CO* positively regulates two floral integrators. In Arabidopsis, *CO* activated *SOC1* through *FT* to promote flowering (Rosas et al. 2014; Lee and Lee 2010). In this study, compared to R2B1-15, *CO* showed a higher expression in R4B1-15 (Fig. 7). Although *CO* transcription regulation was affected by blue light treatment, *CO* was not only positively regulated by *CRY2* and *PHYA*, but also negatively regulated by *PHYB* (Valverde et al. 2004). The lower expression under a high proportion of blue irradiation might result from the co-regulation of the cryptochrome receptor and phytochrome receptor. In Arabidopsis, blue light could induce *SOC1* and *FT* expression to promote flowering (Hori et al. 2011). In petunia plants, blue light induces *FBP28* expression, the orthologous gene of *SOC1* (Fukuda et al. 2011). *SOC1* is one of the direct targets of *AGL24* and is up-regulated by *AGL24*. In Arabidopsis, *SOC1* and *AGL24* are able to up-regulate each other’s expressions, both of them are MADS-domain-containing transcription factors that determine flowering time (Michaels et al. 2003). In this study, *FT* showed a higher expression in R2B1-10 and R2B1-15 than in R4B1-10 and R4B1-15 (Fig. 8b), which suggested that higher proportion of blue light irradiation accelerated cucumber flowering through inducing *FT* expression. Two *SOC1* orthologous genes and seven *AGLs* (only *AGL19* showed different expression) orthologous genes were found in cucumber shoot apices. *SOC1* and *AGL19* displayed the same expression pattern, with increased expression in R2B1-10 and R2B1-15 treatments (Fig. 8b). All these results suggested that a higher proportion of blue light induced cucumber flower bud formation and promoted flowering time through up-regulating expression of these flowering regulation factors.

### Light quality affects flower sex differentiation of cucumber seedlings through regulating plant hormone signaling transduction

In this study, we found that under a higher proportion of blue light (R2B1) treatment, the first female flower node declined significantly and the number of female flowers within fifteen plant nodes increased markedly (Table 1). Transcriptome analysis of cucumber seedlings under different light treatment by RNA-seq technology showed that there were more DEGs under R2B1 treatment. These DEGs were mostly involved in the hormone signaling pathway (Table 3). This suggested that a higher proportion of blue light regulated female flower formation through mediating plant hormone signaling pathways.

Plant sex differentiation is closely related to plant hormones, such as ethylene, which is considered as a potent sex hormone in cucumbers that can induce formation of female flowers (Trebitsh et al. 1987; Rudich et al. 1972; Li et al. 2017). Another example is the gibberellins, which can promote the formation of male flowers via ethylene-dependent and ethylene-independent pathways in cucumber (Hao et al. 2003). ABA can promote male flower development (Zhu et al. 2010), while auxin is able to promote the female tendency of vegetables and induce female flower development through triggering ethylene synthesis (Atsmon and Tabbak 1979; Galun et al. 1962). Exogenous CTK application can turn the grape male flower into a female flower (Chang et al. 1999; Negi and Olmo 1966). JA can regulate gynoecium development (Figueroa and Browse 2015), while BR induces androecium development in maize and inhibits gynoecium development (Hartwig et al. 2011; Makarevitch et al. 2012). In this study, we found that among these DEGs related to plant hormone transduction pathways, the specific DEGs related with IAA formed the higher proportion of DEGs in plant hormone signal transduction, which was followed by the specific DEGs related with CTK, ABA, ETH, BR, JA and SA (Fig. 5).

Ethylene is considered as a potent sex hormone in cucumber that can induce formation of female flowers (Malepszy and Niemirowicz-Szczytt 1991). Ethylene content in shoot apices of gynoecious cucumbers is higher than that of monoecious plants (Rudich et al. 1972; Fujita and Fujieda 1981; Trebitsh et al. 1987). Treatment with exogenous ethylene or ethylene-releasing reagents can increase the numbers of female and bisexual flowers in monoecious and andromonoecious lines, respectively (Mcmurray and Miller 1968; Iwahori et al. 1969). Until now, the molecular mechanism of ethylene-regulated sex determination of cucumber has been well understood. Ethylene biosynthetic genes and ethylene signal transduction genes are involved in the sex expression of cucumber (Wang et al. 2010). In the ethylene signal transduction pathway, the receptors, such as ETRs, function as negative regulators, while ERFs (downstream components of receptors) act as positive transcription factors to regulate sex determination (Tao et al. 2018; Prescott et al. 2016). In our study, the expression levels of some *ERFs* were increased under R2B1 treatment compared to R4B1 treatment (Fig. 6a). They were probably involved in cucumber sex expression through inhibiting the differentiation into males and promoting the differentiation into females. However, this understanding was based on bioinformatics analysis. The precise roles of *ERFs* in cucumber flower development remained unclear and should be verified in future studies using advanced physiological and molecular techniques. *CTR1* is a negative regulator of ethylene signaling, while *CsCTR1* expression gradually declined during male flower development and increased during female flower development (Bie et al. 2014). In our study, *CsCTR1* expression increased on the 5th day after a higher proportion of blue light treatment.

Ethylene plays a key role in plant sex determination, with several research results proving that there is communication between auxin and ethylene during plant development (Trebitsh et al. 1987; Gao et al. 2015; Wang et al. 2018). In our study, we found a considerably number of transcription factors involving in auxin signaling pathway altered their expression levels under R2B1 treatment (Fig. 6). IAA may play a key role in effect of light quality on sex differentiation in cucumber. IAA has been considered as a transcription factor activated by phytochrome, which participates in signaling transduction. During the early development of plants, there are several reactions between phytochrome and auxin, such as auxin homeostasis promoted by light (Zhang et al. 2014c). Auxin is an important regulator in the development of male flowers (Sakata et al. 2010). The auxin synthesis pathway can be regulated by aldehyde oxidases, which have been identified in cucumber (Brown and Purves 1978). Aldehyde oxidases are involved in the conversions of indole-3-acetaldehyde to IAA (Mano and Nemoto 2012). Higher activity of aldehyde oxidase has also been measured in the auxin-overproducing superroot1 (sur1) mutant of Arabidopsis (Seo et al. 1998). *Aldehyde oxidase* was reported to regulate auxin and ABA biosynthesis pathway (Koshiba et al. 1996; Seo et al. 1998, 2000). In our study, the expression of *Aldehyde oxidase* (*Csa4G269130*) was down-regulated in R2B1 samples (Additional file 5: Table S2), which suggested that the auxin synthesis or ABA synthesis in cucumber seedlings flowers was low under a higher proportion of blue light. This result was contradicted against the finding that more female flower were formed in the R2B1 samples which might result from higher auxin content. Meanwhile, it was also contrary to the most up-regulated auxin responsive genes *SAURs* (Fig. 6). While the expression of auxin transporting gene LAX1 and PIN1 were also altered indicating that the auxin transport was changed inside the cucumber flower. Even though the low biosynthesis might be found in the flowers, the higher transportation might also cause a high auxin content in the flower tissues. However, auxin content detection and gene function analysis experiments should be performed to confirm this hypothesis.

Gibberellins, a class of tetracyclic diterpenoid phytohormones, can promote the differentiation of cucumbers into male flowers. GA production in andromonoecious cucumbers is higher than that in gynoecious and monoecious plants (Junior et al. 1972). Exogenous GA_3_ application can increase the ratio of males to females in monoecious cucumbers and induce the formation of male flowers in gynoecious plants (Wittwer and Bukovac 1962; Pike and Peterson 1969). In our study, we identified two DEGs involved in the GA synthesis pathway. Ent-copalyl diphosphate synthase (CPS) is limiting for ent-kaurene production in the first portion of the GA synthesis pathway (Prisic and Peters 2007). *CsCPS1* was down-regulated in R2B1-5 and up-regulated in R4B1-15 (Fig. 6), which suggested that a high proportion of blue light reduces levels of GA in cucumber seedling flowers through breaking down the *CPS1* gene expression. GA2-oxidase (GA2ox) is also a key gene for GA synthesis pathway. It catalyzes catabolism and inactivation of bioactive GAs or their precursors (Schomburg and Amasino 2003; Lester et al. 1999; Thomas et al. 1999). In our study, *CsGA2ox2* was up-regulated in R2B1-10 samples (Fig. 6), which suggested that a high proportion of blue light reduces active GA levels through inducing the expression of *CsGA2ox2*. In addition, the GA signaling pathway is involved in stamen and anther development in hermaphroditic plants, such as Arabidopsis and rice (*Oryza sativa*) (Aya et al. 2009; Plackett et al. 2011; Sun 2011). However, in this study, we did not identify any DEGs involved in GA signaling pathway.

PIF, phytochrome interaction factor, plays a central role in light signal transduction mediated by phytochrome (De and Prat 2014). PIF3 can activate the transcription factor *EIN3* directly, which was involved in the ethylene signaling pathway (Zhong et al. 2012). *COI1*, *JAZ*, *MYC2* and *JAR1* involved in the JA signaling pathway affects the light signal response (Kazan and Manners 2011). *PHYB* negatively regulated ABA accumulation through mediating light signals, while *PHYA* positively regulated ABA signaling pathway under far-red light treatment (Gu et al. 2012; Mach 2014). Thus, it was seen that light could regulate plant sex differentiation through mediating hormones by light receptors. The phytohormones, auxin, BR, CK, ETH, GA and JA, influenced sex differentiation in flowering plants (Durand and Durand 2010; Louis and Durand 1978; Chailakhyan 1979; Irish and Nelson 1989). Our results showed that the expression of some key genes involved in ABA, auxin, CK, ETH, GA and JA biosynthesis pathways significantly changed during development and sex expression, which suggesting that these hormones might participate in these processes. Thus, it is interesting to investigate how these hormones interact with one another to regulate the abortion of male flowers in gynoecious plants.

### Light quality affects flowering time of cucumber seedlings through regulating transcription factors

A total of 433 transcription factors have been identified by RNA-seq technology. Among them, ethylene transcription factor accounted for 24.02%, *MYB* transcription factor accounted for 20.79%, *bHLH* transcription factor accounted for 16.17% and *WRKY* transcription factor accounted for 10.62%.

Most studies have shown that *MYB*, *bHLH* and *WRKY* transcription factors play an important role in biotic stress and abiotic stress (Oh et al. 2012; Li et al. 2010; Jiang and Yu 2009), but some studies proved that they were also related to flower development (Wang et al. 2011; Luo et al. 2013; Zhang et al. 2014b). In Arabidopsis, JA mediates the transcription factors *MYB21* and *MYB24* to regulate stamen development (Song et al. 2011), while *MYC5* regulated stamen development by activating JA signaling pathway through inducing *MYB21* expression. *PIFs* belongs to *bHLH* family of transcription factors, which interact with activated Pr to initiate phytochrome signaling transduction. In Arabidopsis, *PIF4* accelerated flowering by activating the *FT* gene (Kumar et al. 2016). *WRKY* family members are key factors for the ABA response pathway (Rushton et al. 2012). It had been shown that *WRKY* transcription factors regulated plant flowering. *WRKY25* accelerated flowering through negatively regulating *AP1* directly or indirectly (Wang et al. 2011). In pea, *GsWRKY20* promoted flowering by regulating the flowering related genes *FT*, *SOC1* and *CO* (Luo et al. 2013).

## Materials and methods

### Plant materials and treatments

LED light produced by Kedao technology corporation (Huizhou, China) (Red light: 650–660 nm and Blue light: 450–460 nm) was chosen for this study. Cucumber (*Cucumis sativus* L. cv. Huaqing No. 5) seeds were cultivated on a sponge block with a Yamasaki culture solution. When cotyledon appeared, we placed these seedlings under light conditions as follows: 300 μmol/m^2^/s, 12/12 (light dark) at 25 °C and a relative humidity of 70–80%. There were 2 treatments with ratios of red to blue being 4:1 (R4B1) and 2:1 (R21B), respectively. Each treatment lasted 20 days. As the flower primordium appeared 10 days after light treatment, the shoot apices and leaves after 5, 10 and 15 days of light treatments were collected immediately flash frozen in liquid nitrogen and stored at − 80 °C until use. Two biological replicates of shoot apices were used for RNA-seq analysis and real-time RT-PCR. After 20 days of treatment, 30–40 seedlings from each treatment were transplanted into the greenhouse of South China Agriculture University with a relative humidity ranging from 70 to 85% and 28 °C/20 °C (14 h/10 h) day/night temperature for further flowering time analysis.

### cDNA library construction and illumina sequencing

The cucumber shoot apices stored at − 80 °C were provided to Guangzhou Gene Denovo Biological Technology Co., Ltd. (Guangzhou, China). RNA isolation and RNA-Seq library preparation and sequencing were carried out by Guangzhou Gene Denovo Biological Technology Co., Ltd. (Guangzhou, China). In brief, the workflow of library construction for transcriptome analysis was as follows: After RNA was collected, poly(A)-containing mRNA was purified using oligo(dT) magnetic beads. Following this, the mRNAs were fragmented and cDNA was synthesized using a random hexamer, DNA polymerase I and RNase H. The double-stranded cDNAs were purified and ligated to adaptors for Illumina paired-end sequencing. PCR amplication was done on the purified cDNAs with ligated adaptors. PCR products were separated by 2% agarose gel. The final PCR product chosen for sequencing is 400–500 bp band, which were cut and recovered from the gel. The quality and quantity of the library were verified using an Agilent 2100 Bioanalyzer and ABI real time RT-PCR, respectively (Additional file 6: Table S3 and Additional file 2: Figure S1). The cDNA libraries were sequenced using Illumina HiSeqTM 2500 by Gene Denovo Co. (Guangzhou, China). The libraries of two biological replicates of shoot apices were prepared independently.

### Sequence read mapping and assembly

The original image data produced by sequencing were transferred into sequences (raw reads). The calculating methods for raw reads was conducted according to Cock (2010). To obtain clean reads for further analysis, the raw reads were filtered by removing adaptor sequences, low-quality reads and reads with a percentage of unknown bases (N) of more than 10%. Following this, we used Bowtie to remove the ribosome reads (Langmead et al. 2009). The clean reads were mapped to the cucumber genome assembly (Chinese long) v2 (http://www.icugi.org/cgi-bin/ICuGI/index.cgi) according to Kim et al. (2013) and Trapnell et al. (2012).

### Quantification and differential expression analysis of transcripts

The expression level of each gene was determined by the numbers of reads, which were uniquely mapped to the specific gene and the total number of uniquely mapped reads in the sample. Reads per kilobase of exon model per million mapped reads (RPKM) were calculated according to Mortazavi to estimate gene expression levels. The FDR was used to determine the threshold of the P-value in multiple tests. In our study, a FDR < 0.05 and an absolute value of |log2 (fold change)| > 1 were used as the threshold to determine the significant DEGs.

### GO and KEGG enrichment analysis

To identify putative biological functions and pathways for the DEGs, the Gene ontology (GO) and Kyoto encyclopedia of gene and genomes (KEGG) database were searched for annotation. The methods for GO and KEGG enrichment analysis were according to Young and Mao respectively (Young et al. 2010; Mao et al. 2005).

### Real-time RT-PCR

Total RNA was isolated from 100 mg cucumber leaves and shoot apices using Huayueyang reagent kit (with DNase I step: Pipet the DNase I onto the membrane containing RNA. Incubate at 37 °C for 30 min) according to the manufacturer’s instructions. The RNA quality and purity were verified by Nanodrop 2000 and electrophoresis on 1.0% agarose gels. The first-strand cDNA was synthesized from aliquots of 1 μg of total RNA using PrimeScriptTM RT Reagent Kit with gDNA Eraser (TaKaRa, RR047A) in a reaction volume of 20 μL. The synthesized cDNA was diluted 20 times with sterile water and used as the template for real-time PCR. The reactions were carried out in a Roche LightCycler 480 system with SYBR Premix Ex Taq II (Tli RNaseH Plus) (Takara Bio, Dalian, China). The reactions reagent mix was 5 μL SYBR Premix Ex Taq II, 1.5 μL cDNA template, 0.4 μL each primer (10 μmol/μL), and 2.7 μL nuclease-free water. The amplification program was 95 °C for 30 s and 40 cycles of 95 °C for 5 s and 60 °C for 30 s. Melting curve analyses were performed at the end of 40 cycles (95 °C for 5 s followed by a constant increase from 60 to 95 °C). In addition, TUA was used to normalize expression levels (Wan et al. 2010), and the relative expression of genes was calculated using the 2^−∆∆Ct^ method (Livak and Schmittgen 2001). The results were analyzed by Excel 2010. qPCR reactions in leaves were prepared in biological triplicate. qPCR reactions in shoot apices for RNA-seq validation were prepared in three biological replicates.

Genes used for Real-Time RT-PCR were designed on the NCBI primer blast website (https://www.ncbi.nlm.nih.gov/tools/primer-blast/). The primer quality and efficiency were checked by qPCR on series of diluting cDNA. The expression of 26 genes chosen from RNA-seq were analyzed by real-time RT-PCR in shoot apex samples (R2B1 and R4B1 at 5 days, 10 days and 15 days). Meanwhile, 9 genes were used for checking light signal transduction in leaves (Additional file 7: Table S4).

## Additional files

**Additional file 1: Table S1.** Effects of different light quality on the formation of female flowers and flowering time.

**Additional file 2: Figure S1.** 2% agrose gel result of PCR product for library sequencing.

**Additional file 3: Figure S2.** Correlation between gene expression levels of two biological replicates for each stage.

**Additional file 4: Figure S3.** Expression pattern of 26 genes validated by real-time quantitative PCR (qPCR).

**Additional file 5: Table S2.** Expression data of all the genes in R2B1 and R4B1 samples.

**Additional file 6: Table S3.** List of primers used in the expression studies.

**Additional file 7: Table S4.** Real-time quantitative PCR (qPCR) quality control (QC).
