# Supplementary material for: Comparative RNA-Seq analysis on the regulation of cucumber sex differentiation under different ratios of blue and red light
Source: Bot Stud. 2018 Sep 10;59:21. doi: 10.1186/s40529-018-0237-7 (PMC6131680; doi:10.1186/s40529-018-0237-7)
Supplement: Supplementary file 1 — Additional file 1: Table S1. Effects of different light quality on the formation of female flowers and flowering time. [file 40529_2018_237_MOESM1_ESM.doc]

**Table S1. Effects of different light quality on the formation of female flowers and flowering time.**

| **Treatment** | **First female flower located node** | **The proportion of female flowers in 25 nodes (%)** | **Days from transplanting to first female flowering** |
| --- | --- | --- | --- |
| R2B1  (Red light: Blue light = 2:1) | 9.70 ± 0.70b | 16.00 ± 0.84a | 21.9 ± 0.132b |
| R6G2B1  (Red light: Green light: Blue light = 6:2:1) | 11.30 ± 1.01ab | 13.60 ± 1.06ab | 29.00 ± 0.73a |
| R4B1  (Red light: Blue light = 4:1) | 13.90 ± 1.08a | 12.80 ± 0.80b | 29.70 ± 1.20a |
| Control  (natural light) | 14.10 ± 1.04a | 12.60 ± 1.01b | 29.90 ± 1.41a |

a,b: Statistically significant variations of mean values at different sampling points (ANOVA, p<0.05) were indicated with different letters. Means denoted by the same letter did not differ significantly at p < 0.05.
