# Supplementary material for: Comparative RNA-Seq analysis on the regulation of cucumber sex differentiation under different ratios of blue and red light
Source: Bot Stud. 2018 Sep 10;59:21. doi: 10.1186/s40529-018-0237-7 (PMC6131680; doi:10.1186/s40529-018-0237-7)

**Fig S1. 2% agrose gel result of PCR product for library sequencing.**

5R8156: R2B1 -5 sample repeat 1 (R2B1-5-1); 5R8157: R2B1 -5 sample repeat 2 (R2B1-5-2); 5R8158: R4B1 -5 sample repeat 1 (R4B1-5-1); 5R8159: R4B1 -5 sample repeat 2 (R4B1-5-2); 5R8160: R2B1 -10 sample repeat 1 (R2B1-10-1); 5R8161: R2B1 -10 sample repeat 2 (R2B1-10-2); 5R8162: R4B1 -10 sample repeat 1 (R4B1-10-1); 5R8163: R4B1 -10 sample repeat 2 (R4B1-10-2); 5R8164: R2B1 -15 sample repeat 1 (R2B1-15-1); 5R8165: R2B1 -15 sample repeat 2 (R2B1-15-2); 5R8166: R4B1 -15 sample repeat 1 (R4B1-15-1); 5R8167: R4B1 -15 sample repeat 2 (R4B1-15-2).

Before gel cutting: M:100bp ladder (from top to bottom: 1500; 1000; 900; 800; 700; 600; 500 (brightest band); 400; 300; 200; 100)


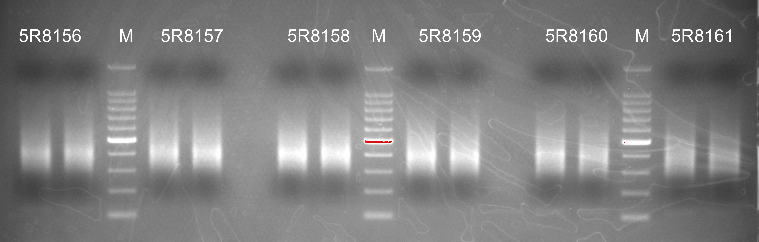


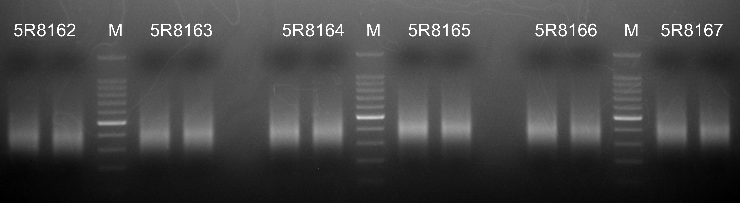


After gel cutting: M:100bp ladder (from top to bottom: 1500; 1000; 900; 800; 700; 600; 500 (brightest band); 400; 300; 200; 100)


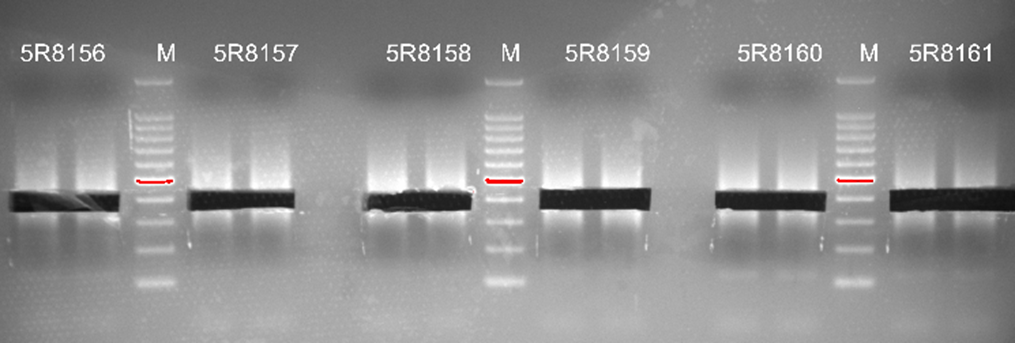


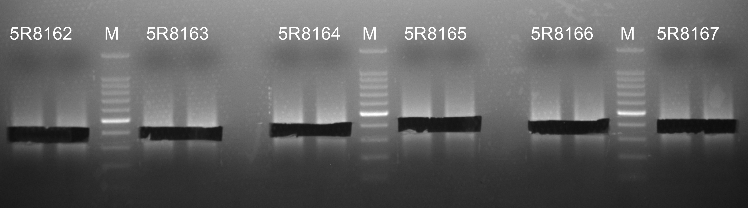

Supplement: Supplementary file 2 — Additional file 2: Figure S1. 2% agrose gel result of PCR product for library sequencing. [file 40529_2018_237_MOESM2_ESM.doc]
