# Supplementary material for: Comparative RNA-Seq analysis on the regulation of cucumber sex differentiation under different ratios of blue and red light
Source: Bot Stud. 2018 Sep 10;59:21. doi: 10.1186/s40529-018-0237-7 (PMC6131680; doi:10.1186/s40529-018-0237-7)
Supplement: Supplementary file 3 — Additional file 3: Figure S2. Correlation between gene expression levels of two biological replicates for each stage. [file 40529_2018_237_MOESM3_ESM.doc]

**Fig S2. Correlation between genes expression levels of two biological replicates for each stage.**

Pearson Correlation Coefficient between (A) R2B1-5-1vs R2B1-5-2. (B) R2B1-10-1vs R2B1-10-2. (C) R2B1-15-1vs R2B1-15-2. (D) R4B1-5-1vs R4B1-5-2. (E) R4B1-10-1vs R4B1-10-2. (F) R4B1-15-1vs R4B1-15-2. (G) 12 samples. R2 >0.8 as the significance cutoffs.


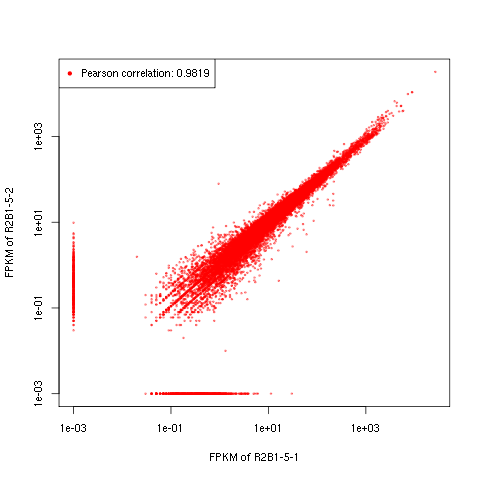

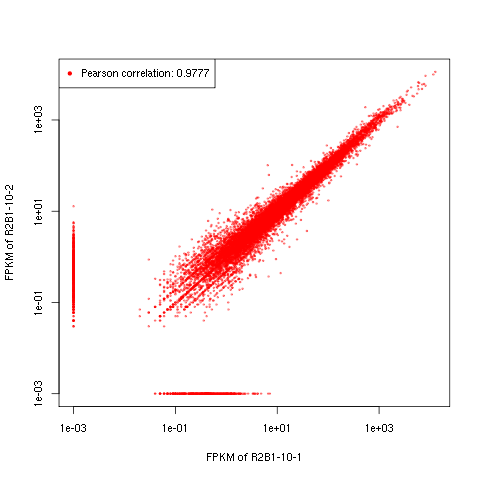


A

B


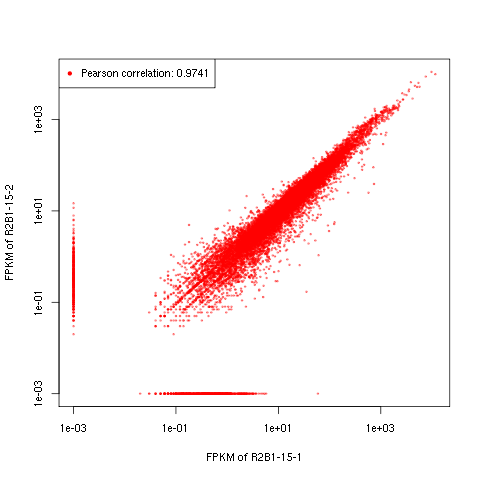

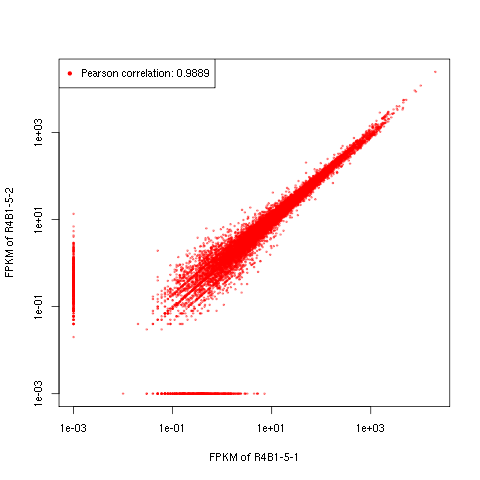


C

D

E

F


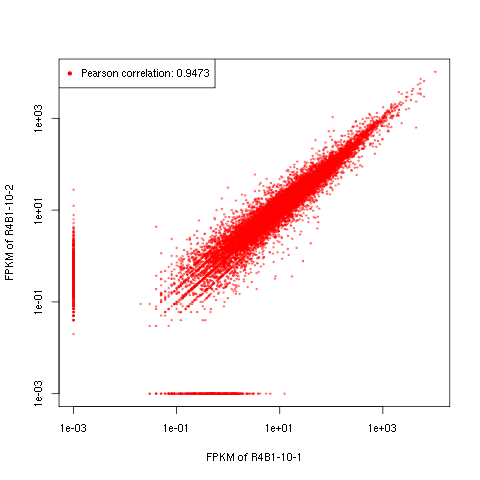

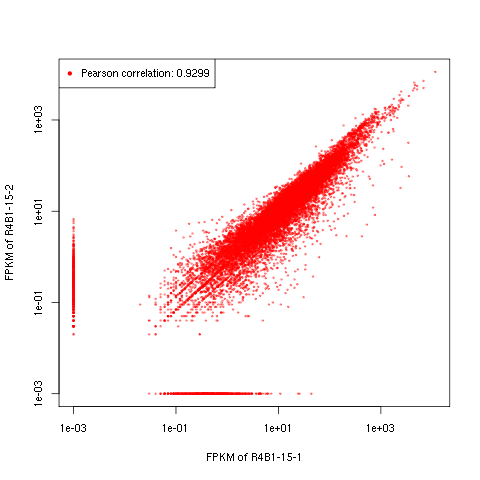


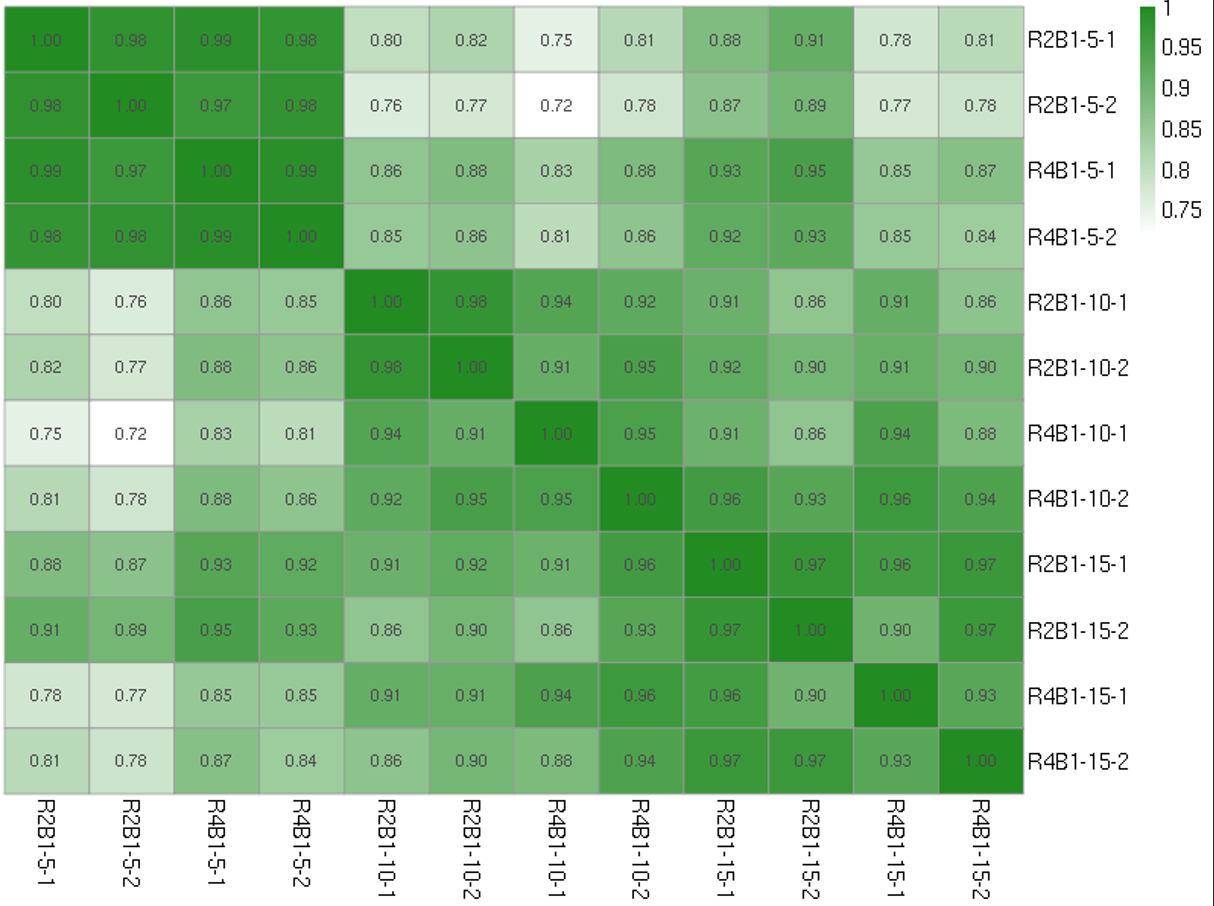


G
