# Supplementary material for: Comparative RNA-Seq analysis on the regulation of cucumber sex differentiation under different ratios of blue and red light
Source: Bot Stud. 2018 Sep 10;59:21. doi: 10.1186/s40529-018-0237-7 (PMC6131680; doi:10.1186/s40529-018-0237-7)
Supplement: Supplementary file 4 — Additional file 4: Figure S3. Expression pattern of 26 genes validated by real-time quantitative PCR (qPCR). [file 40529_2018_237_MOESM4_ESM.doc]

**Fig S3.** **The expression pattern of 26 genes in shoot tips validated by real-time quantitative PCR (qPCR).**

Error bars represent standard deviations of the means of two independent replicates. Statistically significant variations of expression and mean values at different sampling points (ANOVA, p<0.05) are indicated with different letters. R represents Pearson Correlation Coefficient between RNA-Seq and qPCR results. R2 >0.8 as the significance cutoffs.


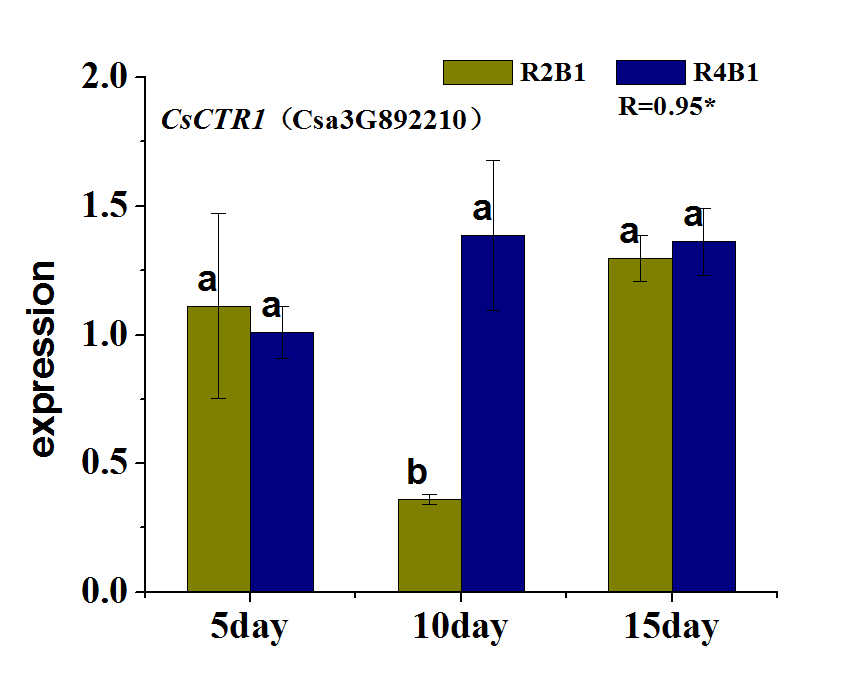

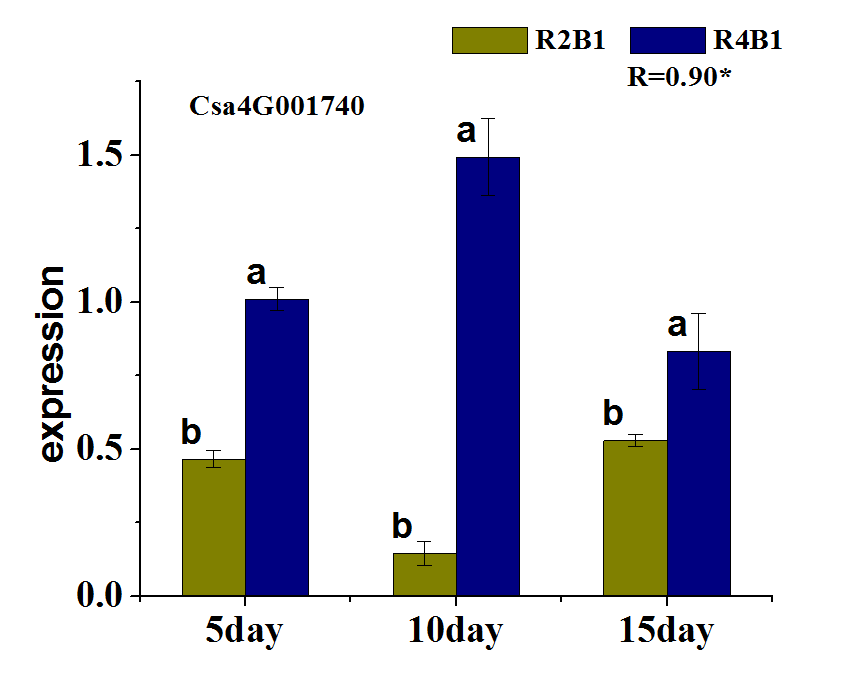


Relative expression

Relative expression


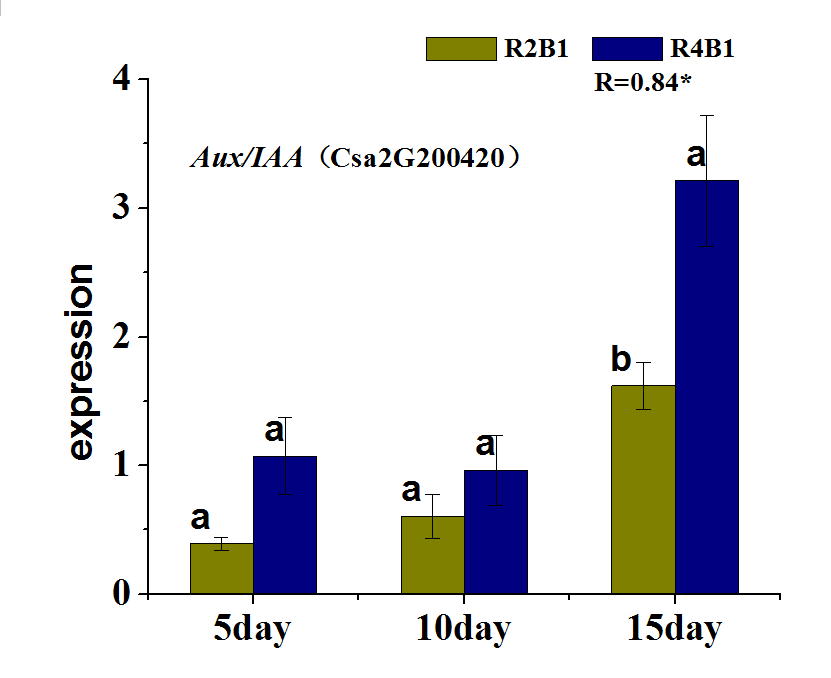

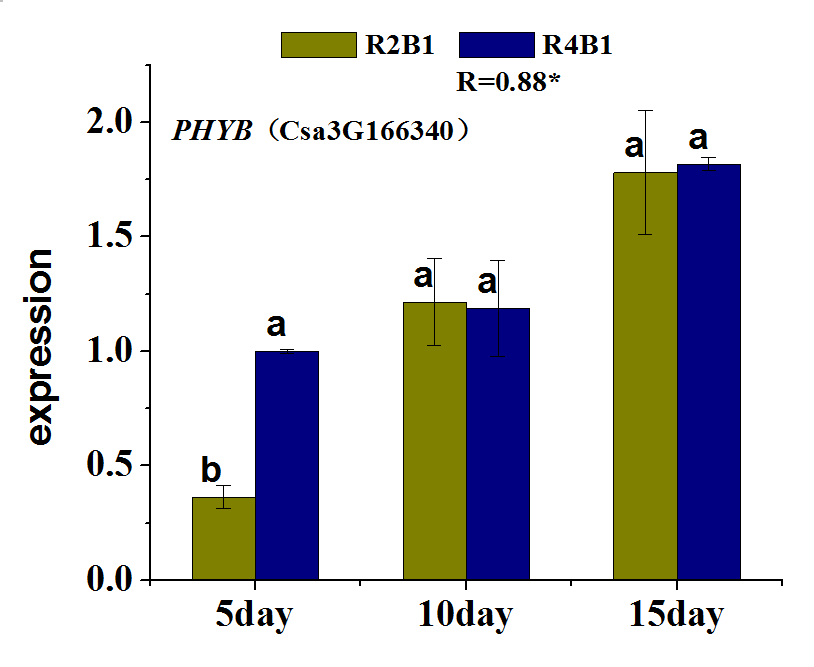


Relative expression

Relative expression


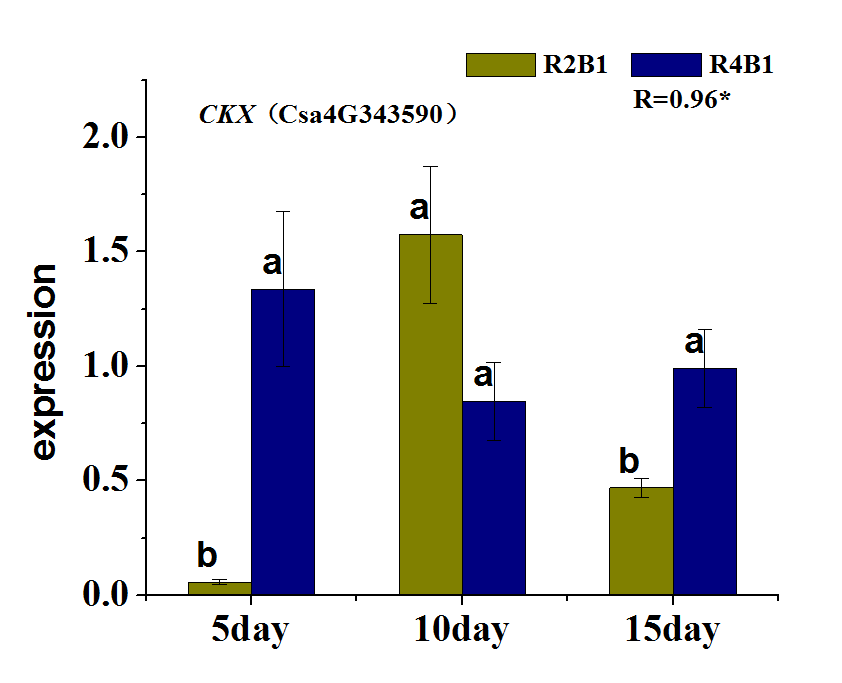

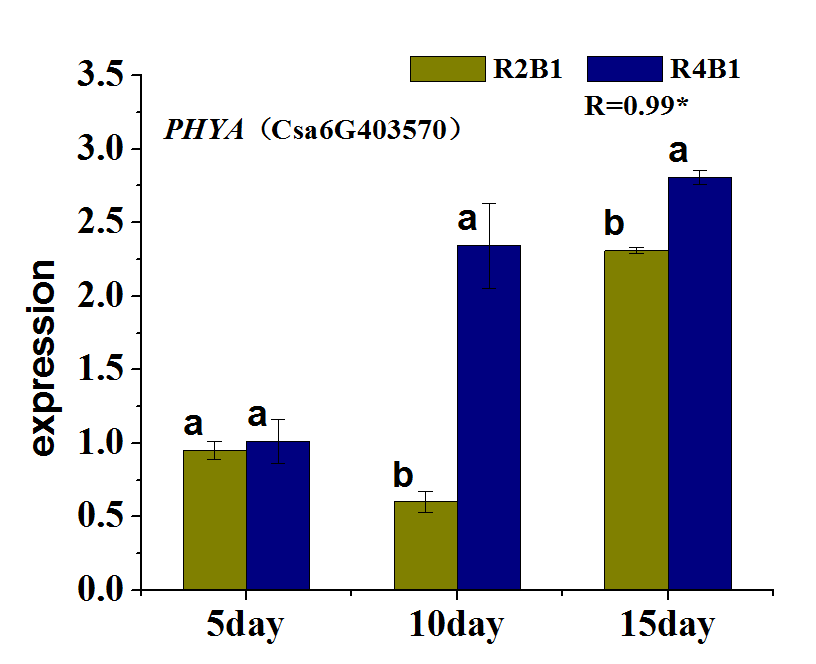


Relative expression

Relative expression


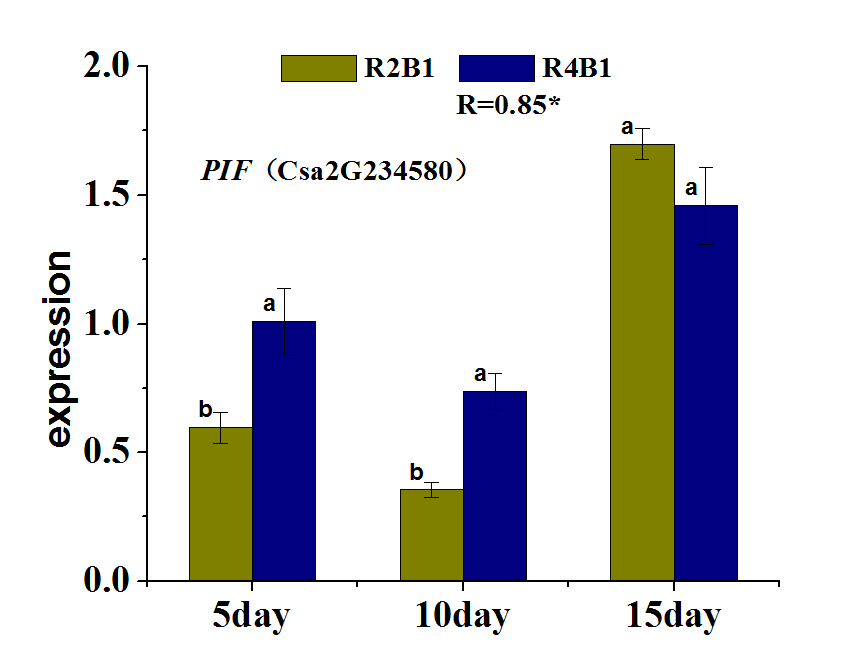

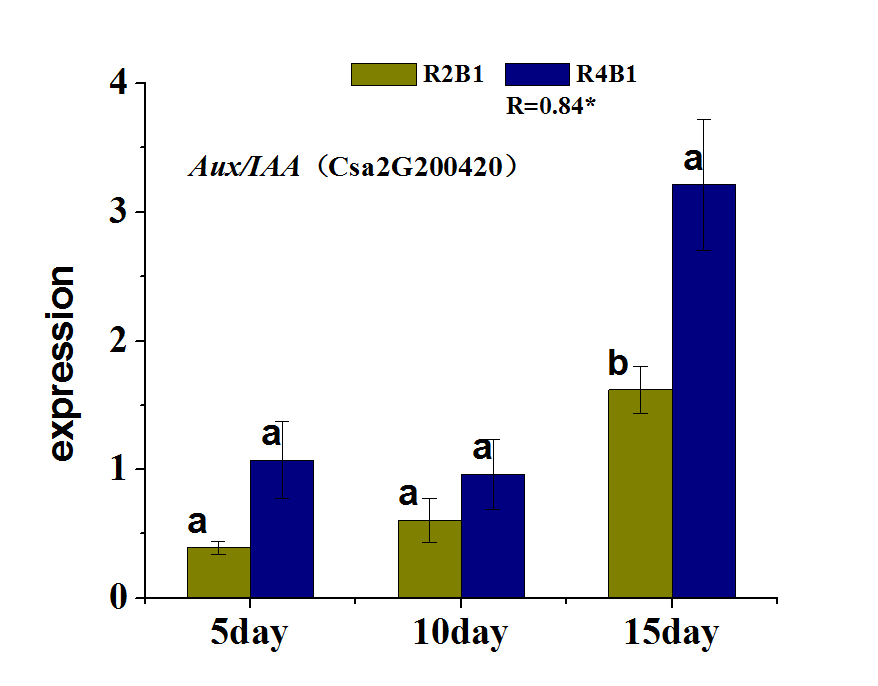


Relative expression

Relative expression


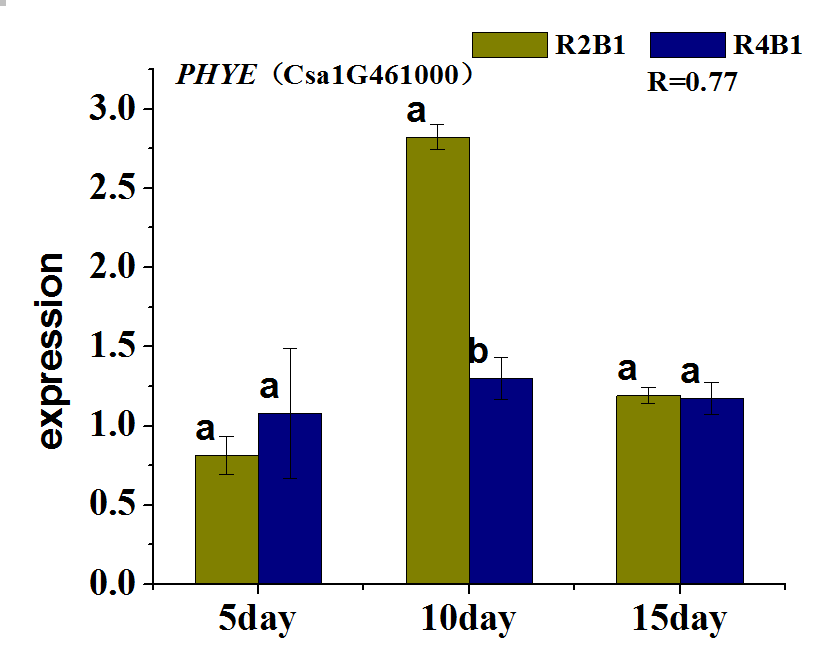

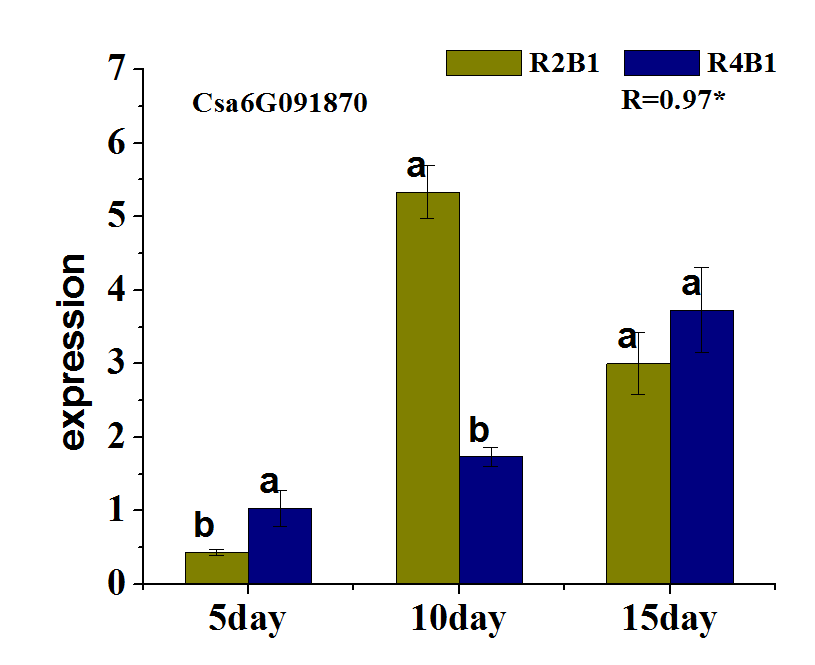


Relative expression

Relative expression


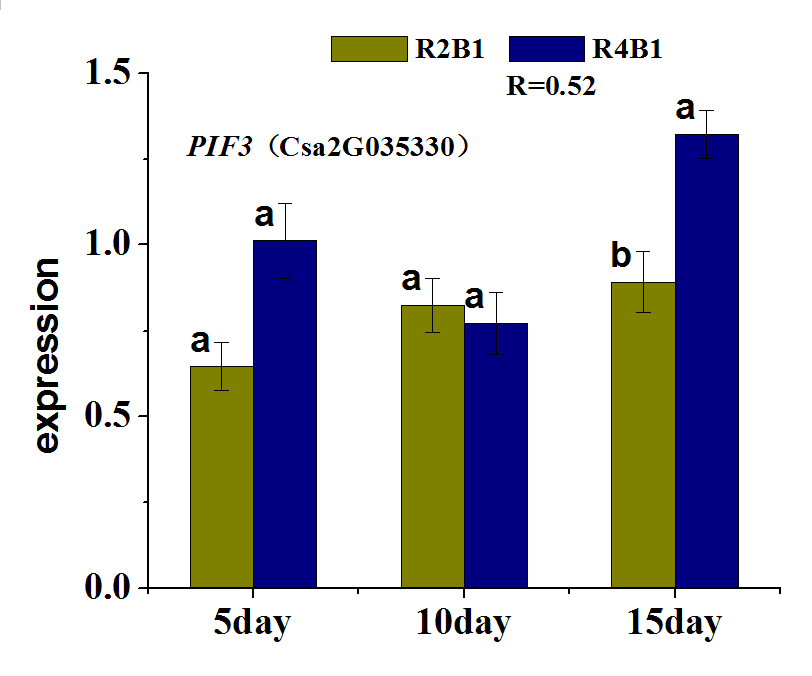

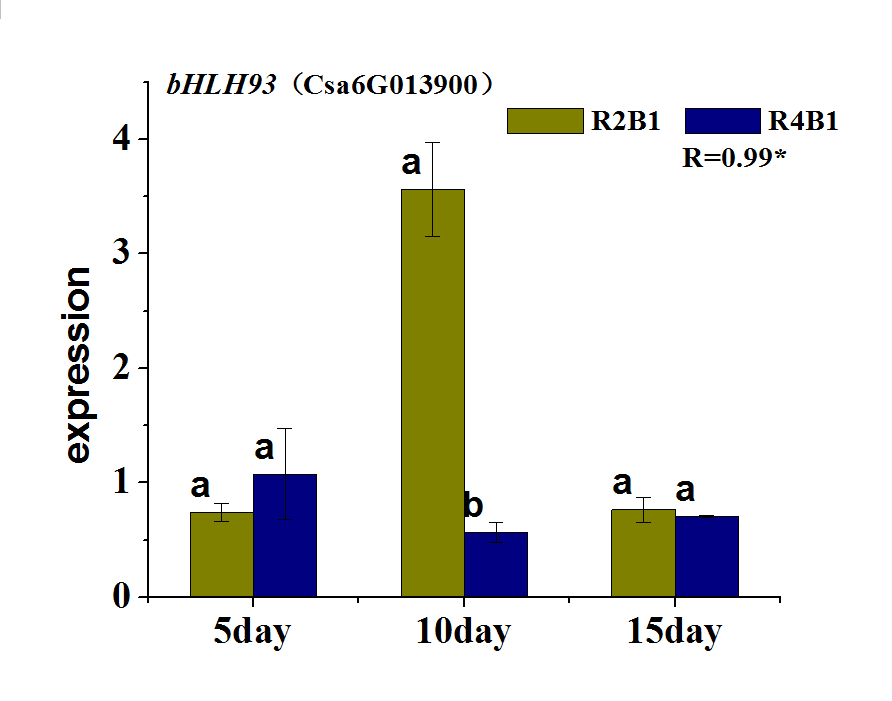


Relative expression

Relative expression


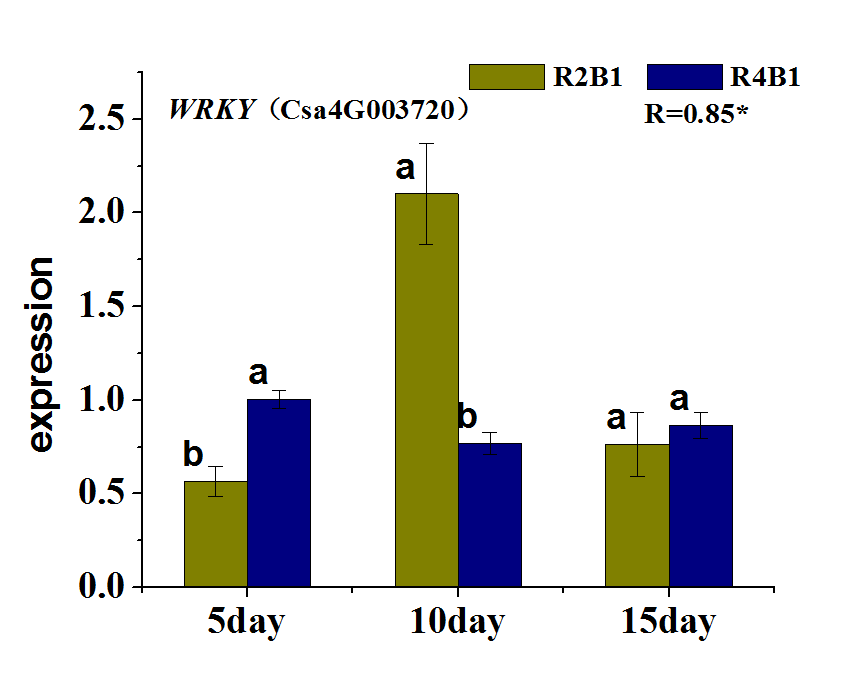

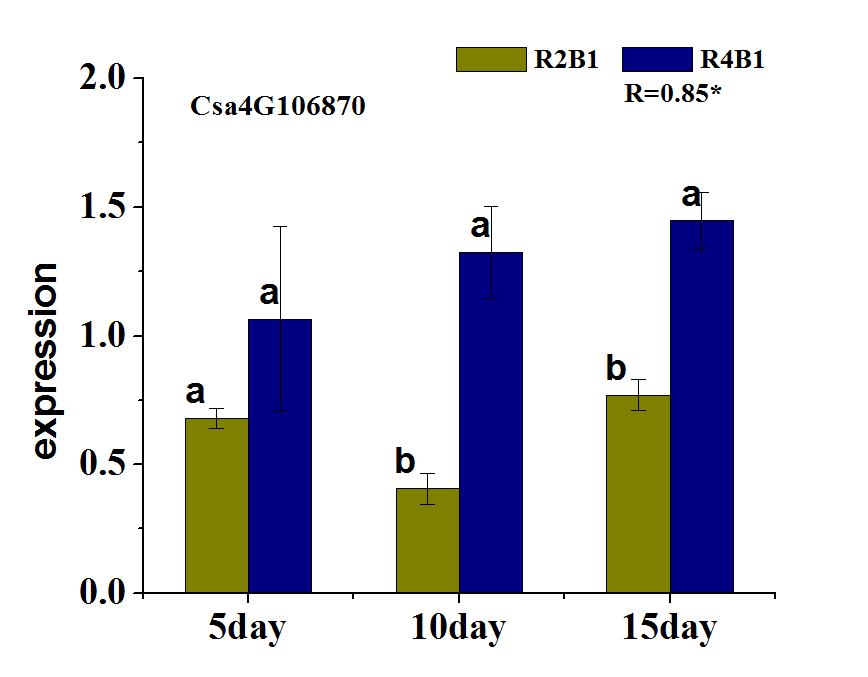


Relative expression

Relative expression


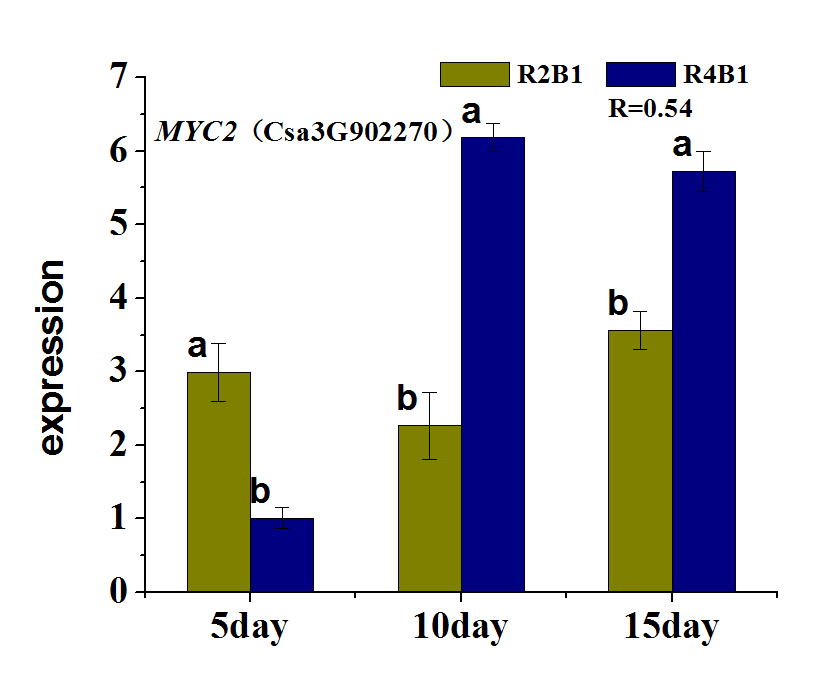

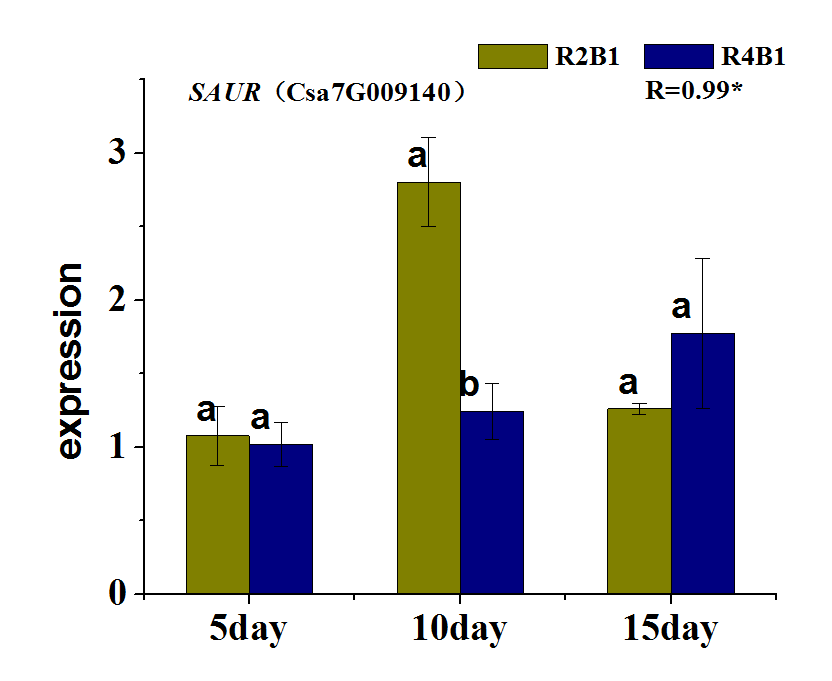


Relative expression

Relative expression


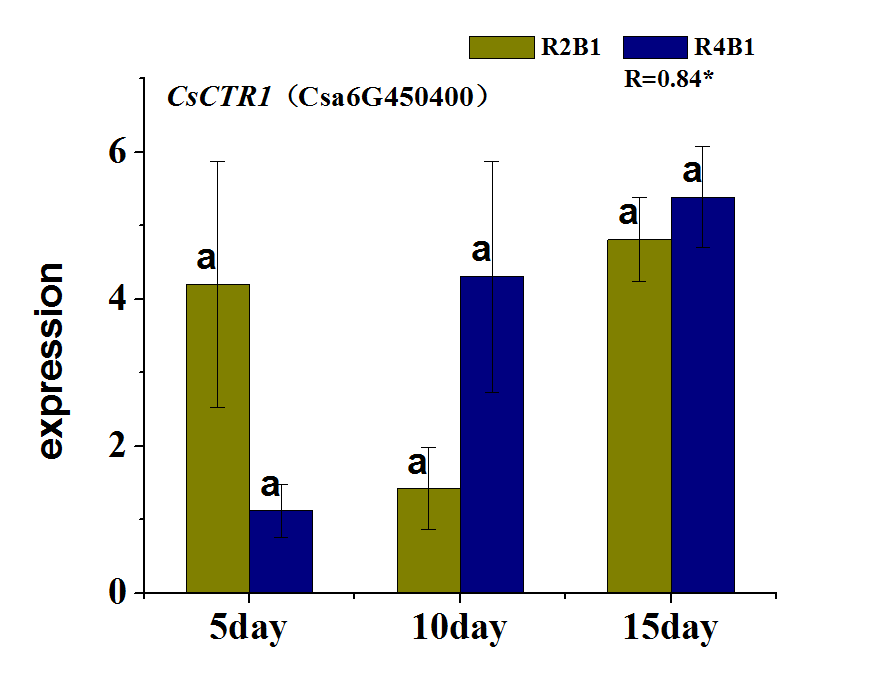

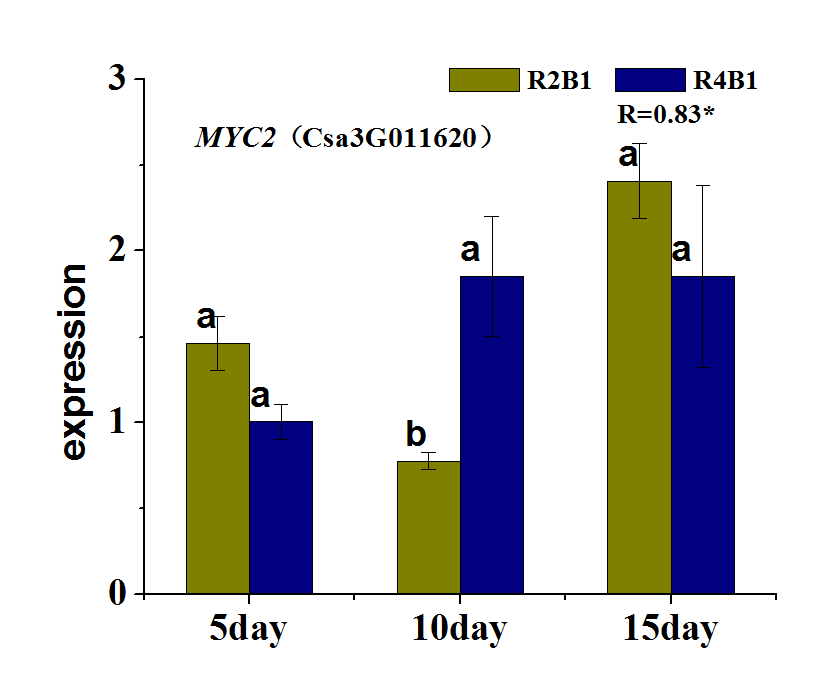


Relative expression

Relative expression


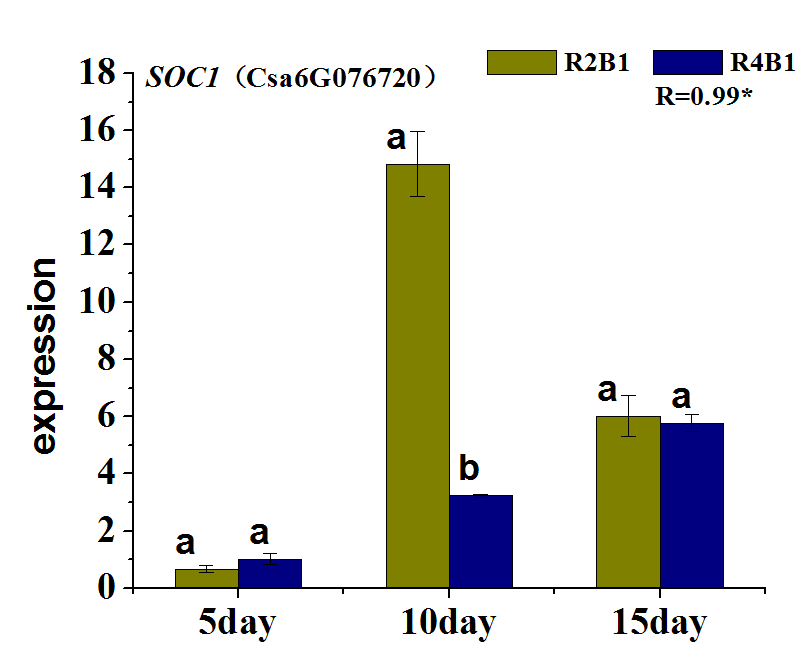

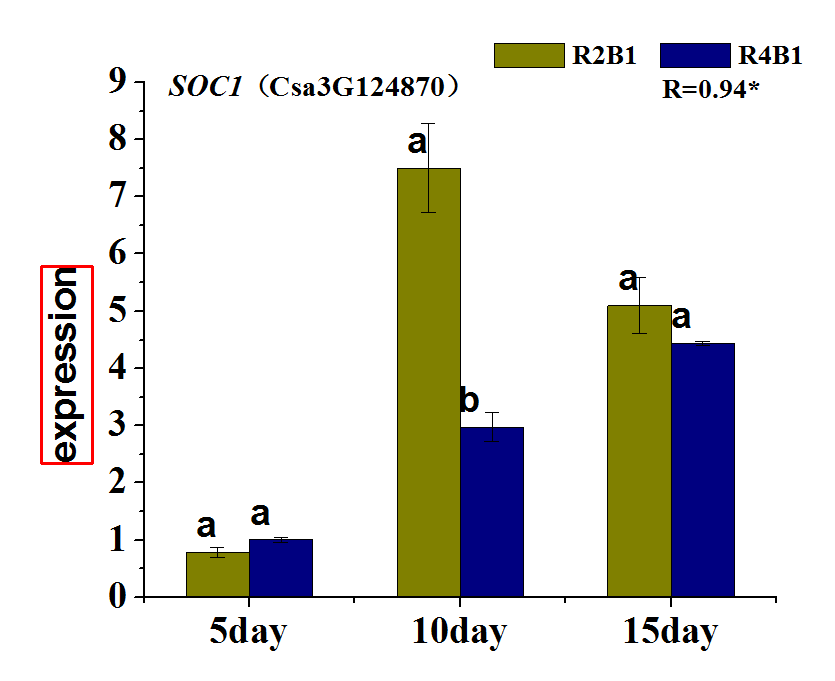


Relative expression

Relative expression


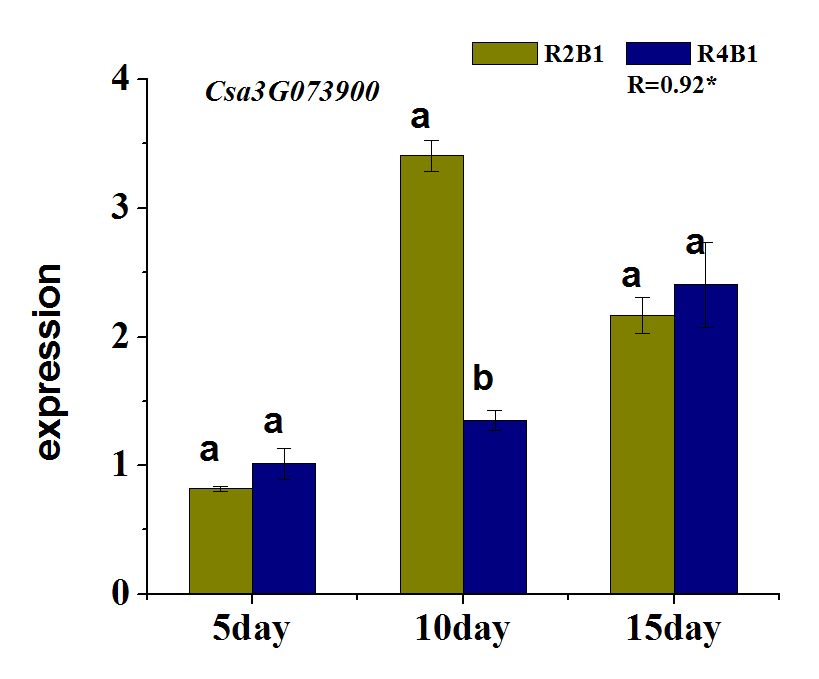

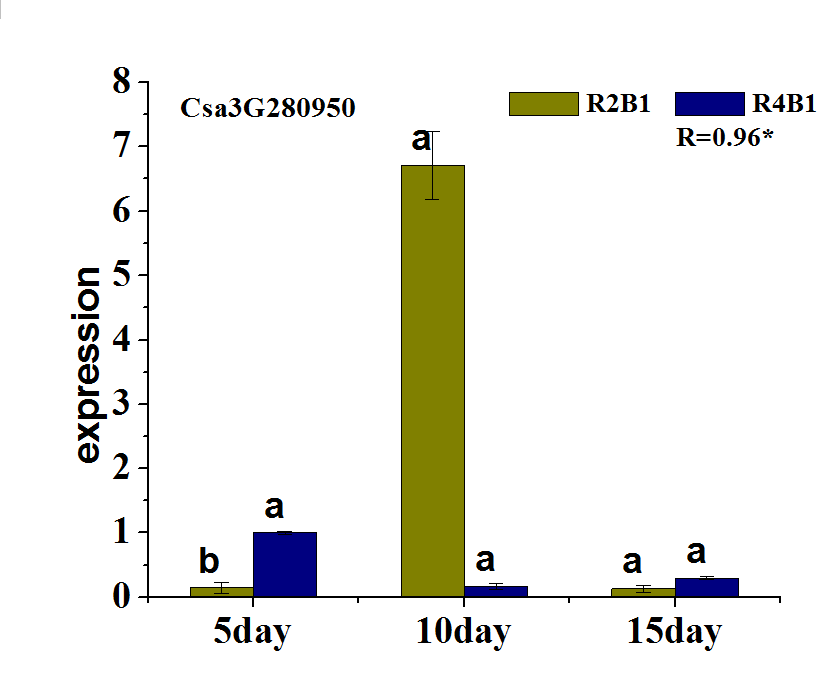


Relative expression

Relative expression


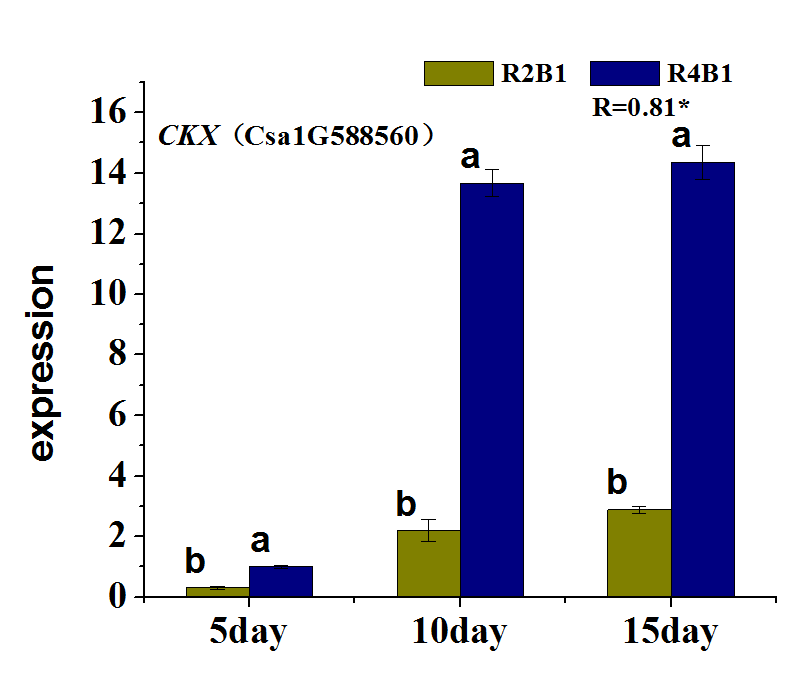

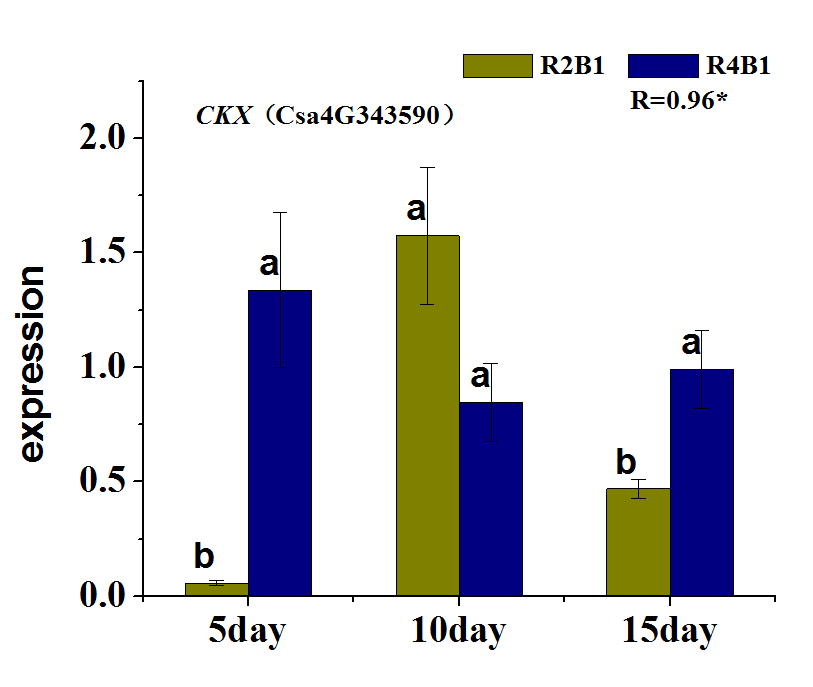


Relative expression

Relative expression


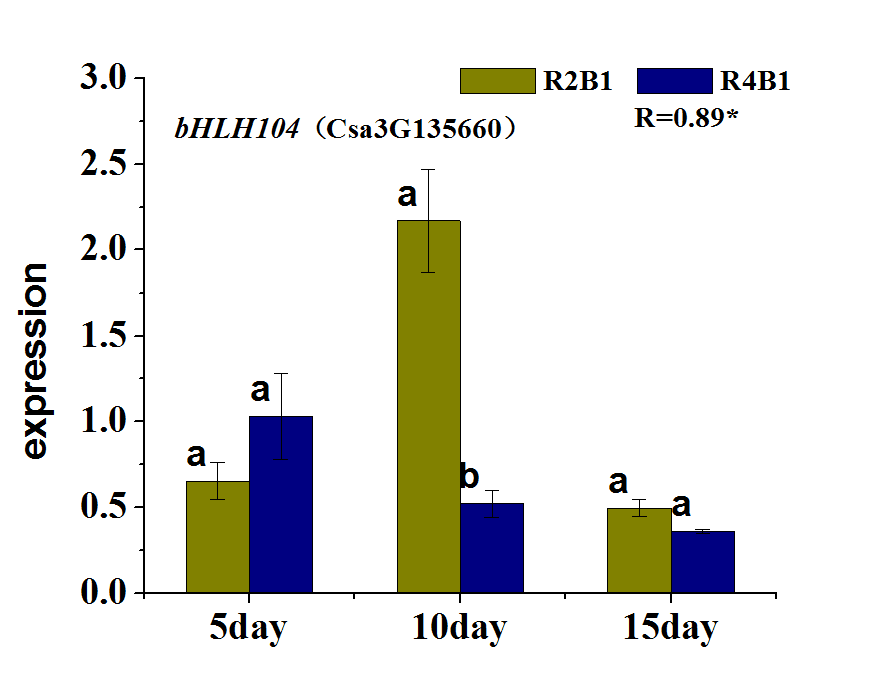

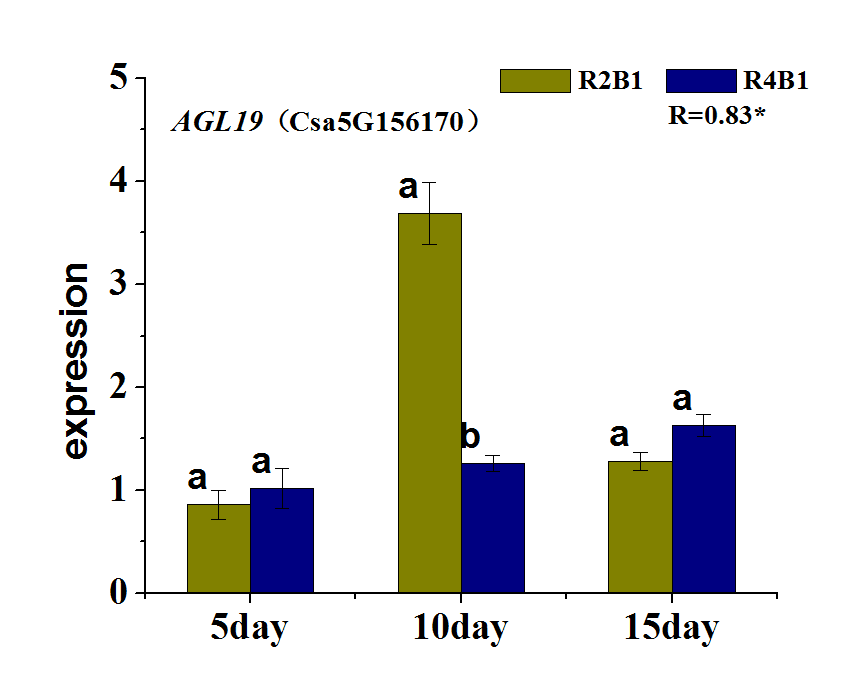


Relative expression

Relative expression
