# Supplementary material for: Comparative RNA-Seq analysis on the regulation of cucumber sex differentiation under different ratios of blue and red light
Source: Bot Stud. 2018 Sep 10;59:21. doi: 10.1186/s40529-018-0237-7 (PMC6131680; doi:10.1186/s40529-018-0237-7)
Supplement: Supplementary file 6 — Additional file 6: Table S3. List of primers used in the expression studies. [file 40529_2018_237_MOESM6_ESM.doc]

**Table S3: Real-time quantitative PCR (qPCR) quality control (QC).**

5R8156: R2B1 -5 sample repeat 1 (R2B1-5-1); 5R8157: R2B1 -5 sample repeat 2 (R2B1-5-2); 5R8158: R4B1 -5 sample repeat 1 (R4B1-5-1); 5R8159: R4B1 -5 sample repeat 2 (R4B1-5-2); 5R8160: R2B1 -10 sample repeat 1 (R2B1-10-1); 5R8161: R2B1 -10 sample repeat 2 (R2B1-10-2); 5R8162: R4B1 -10 sample repeat 1 (R4B1-10-1); 5R8163: R4B1 -10 sample repeat 2 (R4B1-10-2); 5R8164: R2B1 -15 sample repeat 1 (R2B1-15-1); 5R8165: R2B1 -15 sample repeat 2 (R2B1-15-2); 5R8166: R4B1 -15 sample repeat 1 (R4B1-15-1); 5R8167: R4B1 -15 sample repeat 2 (R4B1-15-2).

qPCR quality control (QC)：

| Sample number | Sample name | Corrected molar concentration (nM) | Twin Peaks | Ready for sequencing |
| --- | --- | --- | --- | --- |
| 5R8156 | R2B1-5-1 | 53.11 | No | Yes |
| 5R8157 | R2B1-5-2 | 52.95 | No | Yes |
| 5R8158 | R4B1-5-1 | 58.97 | No | Yes |
| 5R8159 | R4B1-5-2 | 63.1 | No | Yes |
| 5R8160 | R2B1-10-1 | 59.02 | No | Yes |
| 5R8161 | R2B1-10-2 | 68.04 | No | Yes |
| 5R8162 | R4B1-10-1 | 90.38 | No | Yes |
| 5R8163 | R4B1-10-2 | 82.7 | No | Yes |
| 5R8164 | R2B1-15-1 | 98.72 | No | Yes |
| 5R8165 | R2B1-15-2 | 92.56 | No | Yes |
| 5R8166 | R4B1-15-1 | 99.56 | No | Yes |
| 5R8167 | R4B1-15-2 | 93.53 | No | Yes |
