# Supplementary material for: Comparative RNA-Seq analysis on the regulation of cucumber sex differentiation under different ratios of blue and red light
Source: Bot Stud. 2018 Sep 10;59:21. doi: 10.1186/s40529-018-0237-7 (PMC6131680; doi:10.1186/s40529-018-0237-7)
Supplement: Supplementary file 7 — Additional file 7: Table S4. Real-time quantitative PCR (qPCR) quality control (QC). [file 40529_2018_237_MOESM7_ESM.doc]

**Table S4: List of primers used in the expression studies.**

| Gene locus | Forward primer（5'-3'） | Reverse primer（5'-3'） |
| --- | --- | --- |
| Csa6G450400 | CACAGGTTAAACACGCAGGC | CTGCAGCGTTTGATAGCGTC |
| Csa3G892210 | AATCTGTTGCTGGAACGCCT | TTCTGGAAAGCGACAGCTCC |
| Csa3G073900 | GTCAACGCTCCAGATCCCTT | ATCGCTATGGCAATCCTCGG |
| Csa4G370550 | GGGTCTTATTCAACGCCGGA | GGCGGAAGTGACTGTTGTTG |
| Csa6G410650 | TTTACCTGGCGAGGTTGGTT | AGTTGGCAGTTGTTGTAGTCCA |
| Csa7G413380 | AACCCCTTCGGTTATGGCTG | ACAACATGGCTGAAAGTAAGAGG |
| Csa7G009140 | GGCGGTGACGACGAATAATG | TCTTCGGTGCAAGGAATCGT |
| Csa2G258780 | TCTCCTCAATGCCAAGCAAGT | TAGTGGAACCACAAACCGCT |
| Csa2G200420 | GCAGTGGTGGAGATCAGACC | GCAGGATCCTTGGAGCAGAG |
| Csa4G343590 | TTTCAGCTGGAGAGCTGTGG | CTGATTTGGGGTCCATGCCT |
| Csa1G588560 | CGGTGGTGAACAGATGTGGA | GATTCCGGCATTCGACAACG |
| Csa6G013900 | ACTCTCATTCTCTAAACAAGGCGT | CTACCCTTTCCACTGTTACCTGC |
| Csa3G011620 | TGGATTTCGGGAACTACCGC | TGCTGGAAGGAACAGTGGTG |
| Csa3G902270 | GTTACCGACACCGAGTGGTT | AAGAGCTCGCAAGTCGATCC |
| Csa4G106870 | TGGTTCCACAACTTGGCGAT | TAAGACGCAGGAAACGCAGT |
| Csa3G124870 | TACGACGTTGAAGAGGAGCG | GGATCTGGACTTGGGAGGTC |
| Csa6G076720 | CCCTGGATCCTCCGTTTGTC | TCAAGGGAGCAAGATCCCAG |
| Csa5G156170 | GCTTGCACTACCCAGCTACT | AGTGCTGCATTTTCTTCCAGC |
| Csa7G031720 | GCTTGCACTACCCAGCTACT | CCCTCAATGCAGACCCACAT |
| Csa3G135660 | CCCGACGCCGACAATCTTAT | CGCCCTCTTCCTGGAACAAT |
| Csa1G461000 | AGTGCAGTATGCGCCTGTT | CCCACAACAAGCCCACAAAATC |
| Csa4G003720 | TGAAGGTCCACCTCCTGATT | GGGACCTGGATGGTTATGGC |
| Csa6G403570 | TCCTGGTGCATTGCAAGACT | CACCCACTAGGCAAGGACTG |
| Csa3G166340 | AGTTACAAGCACTCCCTGGC | TGGCTCTAAGTCAGGCCTCT |
| Csa2G035330 | TGGTCAAGGACTTCCCATGC | GAGCTACAGGCTGCAGTTGA |
| Csa2G234580 | CCCTCTTCGTGATGCCCAAT | AGGGTTTTAGCGGAGAAGCC |
| Csa1M023050 | GTTCCTTTGCCAATCCTGCG | TGGAGGGAGATGATCGGTGG |
| Csa3M889670 | CAACTGCTCCGACACAGACT | CAACCACAAACTGCTCCGTG |
| Csa1M651710 | ATTGGTGGGACCGATCTTCG | CTCTTGCCCAAAGGTTGCAC |
| Csa4G000580 (TUA) | CTCCCTCCTTTTGGAGCGTT | GAAGCACAGCAACGTCAGTG |
